# Lipidomics study of plasma from patients suggest that ALS and PLS are part of a continuum of motor neuron disorders

**DOI:** 10.1038/s41598-021-92112-3

**Published:** 2021-06-30

**Authors:** Estela Area-Gomez, D. Larrea, T. Yun, Y. Xu, J. Hupf, F. Zandkarimi, R. B. Chan, H. Mitsumoto

**Affiliations:** 1 Departments of Neurology, Columbia University Medical Center, Room P&S 4-443, 630 West 168th Street, New York, NY 10032 USA; 2 Eleanor and Lou Gehrig ALS Center, Columbia University Medical Center, New York, NY 10032 USA; 3 Pathology and Cell Biology, Columbia University Medical Center, New York, NY 10032 USA; 4 Biological Sciences, Columbia University Medical Center, New York, NY 10032 USA

**Keywords:** Diagnostic markers, Amyotrophic lateral sclerosis

## Abstract

Motor neuron disorders (MND) include a group of pathologies that affect upper and/or lower motor neurons. Among them, amyotrophic lateral sclerosis (ALS) is characterized by progressive muscle weakness, with fatal outcomes only in a few years after diagnosis. On the other hand, primary lateral sclerosis (PLS), a more benign form of MND that only affects upper motor neurons, results in life-long progressive motor dysfunction. Although the outcomes are quite different, ALS and PLS present with similar symptoms at disease onset, to the degree that both disorders could be considered part of a continuum. These similarities and the lack of reliable biomarkers often result in delays in accurate diagnosis and/or treatment. In the nervous system, lipids exert a wide variety of functions, including roles in cell structure, synaptic transmission, and multiple metabolic processes. Thus, the study of the absolute and relative concentrations of a subset of lipids in human pathology can shed light into these cellular processes and unravel alterations in one or more pathways. In here, we report the lipid composition of longitudinal plasma samples from ALS and PLS patients initially, and after 2 years following enrollment in a clinical study. Our analysis revealed common aspects of these pathologies suggesting that, from the lipidomics point of view, PLS and ALS behave as part of a continuum of motor neuron disorders.

## Introduction

Amyotrophic lateral sclerosis (ALS) is a motor neuron disorder^1^ that results in progressive muscle weakness and atrophy with fatal outcomes due to respiratory muscle paralysis, mostly within 5 years after diagnosis^2^.

In the last decade, the growing number of genes shown to be associated with familial ALS (fALS) have explained genetic causes in about 5–10% of cases^3^. Yet, the cause of sporadic ALS forms and the mechanism(s) of pathogenesis remain unknown. In addition, the clinical presentation of ALS is quite heterogeneous. Patients often present with symptoms that overlap with other neurodegenerative disorders such as primary lateral sclerosis (PLS), which, in addition to the lack of reliable tests, delays significantly the differential diagnosis of these conditions^4^. Although several clinical diagnostic criteria have been defined^5,6^, the diagnosis of ALS requires tracking the development of distinguishing symptoms for over a year.

PLS is essentially diagnosed by exclusion, once ALS and other motor neuron disorders are excluded after tracking symptoms for 4 years^7^. PLS is a rare disorder characterized by the progressive dysfunction of upper motor neurons with no clinical signs of lower motor neuron degeneration^8^. Contrary to ALS, PLS is not lethal and progresses quite slowly^8^. In addition, while approximately 10% of ALS cases have been linked to mutations in > 20 genes^3^, PLS is sporadic, although genetic mutations in ALS genes can sometimes manifest as PLS^9^. Despite these differences, both diseases have some overlapping clinical symptoms and share many pathological hallmarks^7^, to the extent that PLS has been proposed to represent one end of a continuous spectrum of ALS in which atrophy of the spinal cord or nerve roots does not occur^10^. In support of this view, some ALS cases display rather slow symptomatic progression that could be misinterpreted as PLS^11^. By the same token, some reports have described cases initially diagnosed as PLS that slowly evolved into a classical ALS presentation by manifesting lower motor dysfunction^7^. This underscores the necessity for specific biomarkers that can help understand the underlying biological mechanism(s) driving the differences in the progression and outcome of both diseases.

In the last decade, lipids have emerged as potential biomarkers of neurodegenerative disorders such as AD, PD and ALS. Lipids play a central role in the nervous system, as emphasized by the many neurological pathologies that are triggered by the disruption of lipid pathways^12^. Lipids exert a wide variety of crucial functions for nervous system maintenance, including structural roles (e.g., regulation of ion channels and membrane permeability), molecular signaling (e.g., insulin regulation by phosphatidylinositol), mediation of inflammatory responses (e.g., arachidonic acid), among others^12^. Alterations in any of these processes will change the lipidome of cells, tissues, and the biological fluids that surround them. Therefore, the lipid profile of serum or plasma, cerebrospinal fluid and/or cell culture medium is a valid resource for not only the discovery of new biomarkers, but also for the delineation of specific alterations in one or more metabolic pathways or cellular processes over time^13^.

In this study, we have analyzed the lipid composition of plasma samples from ALS and PLS patients at the time of enrollment into a large prospective study of ALS and PLS (COSMOS; COhort Study of Multicenter Oxidative Stress)^14^ and 2 years later, and compared them with age-matched controls. Our analysis revealed similar lipid alterations versus controls in both disorders that might reflect aspects common to both pathologies; however, from a longitudinal perspective, the progression of some of these changes appears to be more exacerbated in ALS than in PLS. Interestingly, only ALS patients presented with alterations in sphingolipid and glycerophospholipids classes and species. Overall, our lipidomics analysis suggests that PLS and ALS are part of a continuum of motor neuron disorders and supports the contribution of alterations in oxidative metabolism to contribute to the pathogenesis of both of these diseases.

## Results

### Patients

Samples from patients diagnosed with PLS or non-familial ALS fulfilling El Escorial criteria for definite or probable ALS (either spinal or bulbar) were selected from the ALS Multicenter Cohort Study of Oxidative Stress (COSMOS) at Columbia University^14^. For a longitudinal study to characterize the nature and extent of lipidomic changes in ALS, we selected 40 serum samples from definite or probable ALS patients collected not later than 6 months after initial diagnosis (baseline), and compared them to serum samples from these same patients collected 2 years after (follow-up) To get insight into the specificity of these lipid alterations in ALS and/or their association with the progression of the disease, we followed the same criteria to select 28 serum samples from patients diagnosed with PLS from the same COSMOS cohort (Table 1). We also selected 28 control serum samples from sex and aged-matched individuals with no chronic illness, recruited at the same time of ALS and PLS patients from the same community. All samples aliquots had been flash-frozen after collection in the presence of antioxidants (BHT: Butylated hydroxytoluene). A preliminary analysis of lipoprotein composition showed that plasma from ALS and PLS patients presents with significantly higher LDL/HDL and ApoB/ApoA-I ratios (Supp. Fig. 1A), in agreement with previously reported results^15,16^.

**Table 1:** Characteristics of ALS and PLS patients and controls.

	N	Mean	SD	Min	Max
**Control**					
Age	28	64	10.1	42	78
Disease durarion at baseline (months)	28	N/A	N/A	N/A	N/A
ALSFRS-R at baseline	28	N/A	N/A	N/A	N/A
Sex	28	47% Male			
Ethnicity	28	0% Hispanic or Latino; 100% White			
**ALS**					
Age	40	62	8.807	43	77
Disease durarion at baseline (months)	40	3.1	2.8	2	10
ALSFRS-R at baseline	35	38.08	5.09	29	46
Sex	40	57.5% Male			
Ethnicity	40	10% Hispanic or Latino; 80% White, 7.5% Black, 2.5% other			
**PLS**					
Age	28	59	8.81	38	81
Disease durarion at baseline (months)	28	40	25.41	1	96
ALSFRS-R at baseline	28	30.04	5.73	16	42
Sex	28	54% Male			
Ethnicity	28	0% Hispanic or Latino; 100% White			

For lipidomic analysis, we extracted lipid from 0.2 ml serum aliquots that had not been previously thawed. Lipids were extracted from equal amounts of material (0.2 ml/sample) prepared via chloroform–methanol extraction by modified Bligh and Dyer protocol (see “Material and methods” section). Four different aliquots from these baseline (6 months after diagnosis) and follow-up samples (2 years after baseline) were analyzed in triplicate, at two different times over the period of 2 years. We were able to detect more than 500 lipid species from 31 different classes of lipids (Table 2). With spiked internal standards with known concentrations (Supp. Table 1). we were able to calculate concentration of each individual lipid species. After NOMIS normalization (details in “Material and methods” section), we ran principal component analysis (PCA) to detect any outlying samples outside confidence interval of 95%.

**Table 2:** Lipid names abbreviations. List of all lipid classes analyzed. The number of species analyzed per class is indicated in parenthesis.

FC	Free cholesterol	PC	Phosphatidylcholine (25 species)
CE	Cholesterol ester (20 species)	PCe	Ether phosphatidylcholine (25 species)
AC	Acyl carnitine (9 species)	PE	Phosphatidylethanolamine (25 species)
MG	Monoacylglycerol (18 species)	PEp	Plasmalogen phosphatidylethanolamine (25 species)
DG	Diacylglycerol (28 species)	PS	Phosphatidylserine (25 species)
TG	Triacylglycerol (42 species)	PI	Phosphatidylinositol (25 species)
dhCer	Dihydroceramide (12 species)	PG	Phosphatidylglycerol (25 species)
Cer	Ceramide (12 species)	BMP	Bis(Monoacylglycero)phosphate (25 species)
SM	Sphingomyelin (12 species)	AcyIPG	Acyl phosphatidylglycerol (15 species)
shSM	Dihydrosphingomyelin (12 species)	LPC	Lysophosphatidylcholine (9 species)
Sulf	Sulfatide (18 species)	LPCe	Ether lysophosphatidylcholine (9 species)
MHCer	Monohexosylceramide (24 species)	LPE	Lysophosphatidylethanolamine (9 species)
LacCer	Lactosylceramide (24 species)	LPEp	Plasmogen lysophosphatidylethanolamine (9 species)
GM3	Monosialodihexosylganglioside (18 species)	LPI	Lysophosphatidylinositol (9 species)
GB3	Globotriaosylceramide (12 species)	LPS	Lysophosphatidylserine (11 species)
PA	Phosphatidic acid (25 species)		

### Multivariate and machine learning analysis of lipidomics data from ALS and PLS plasma

Due to the fact that the size of samples(n) is relatively smaller than the variables(p), lipid species, dimensionality reduction was necessary. Thus, to possibly narrow down associated lipids species with either ALS or PLS, we first we run an unsupervised Principal component analysis (PCA) model. However, this approach was unable to show a clear separation between control subjects and patients with ALS or PLS (Data not shown).

**Figure 1:**
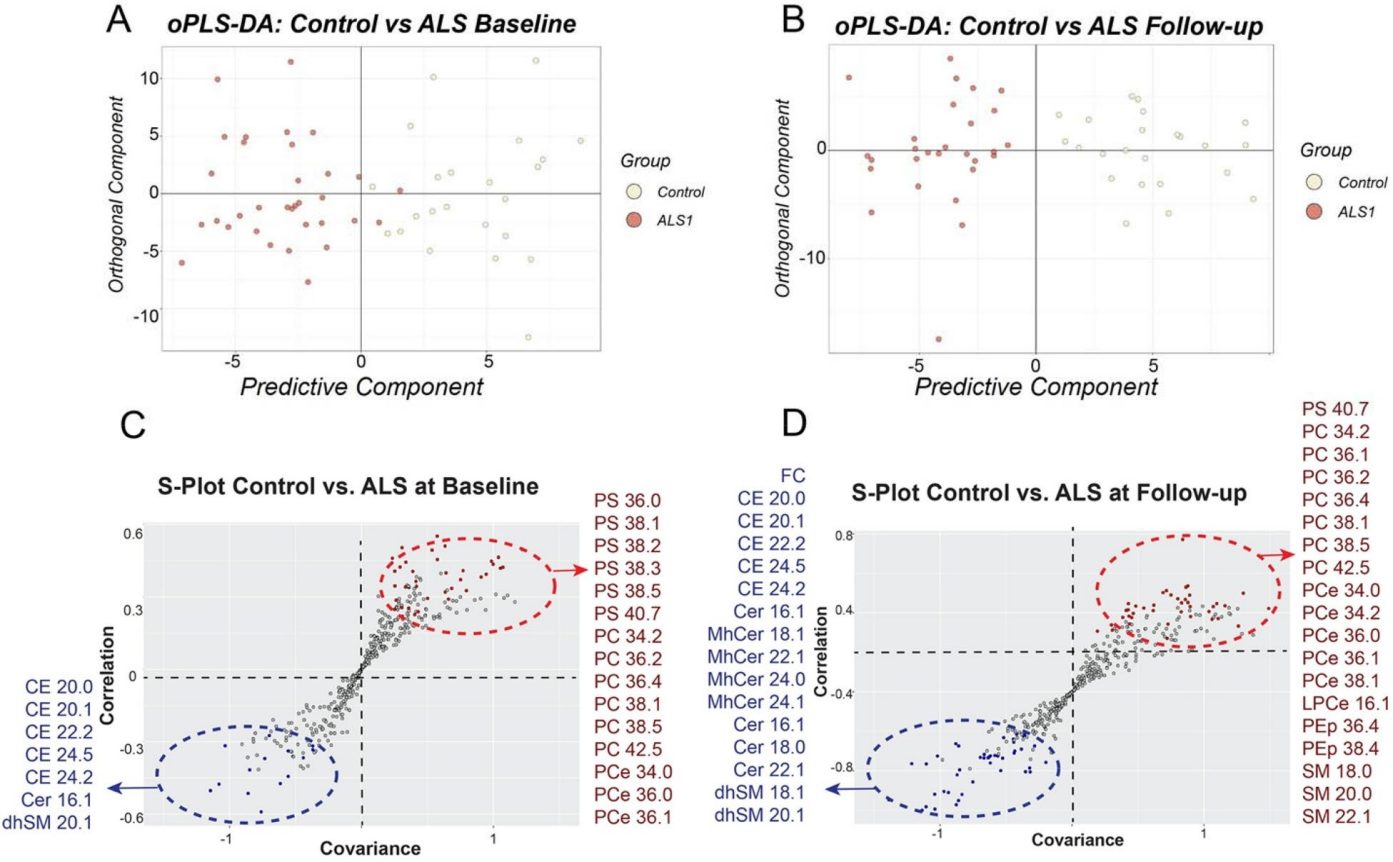
oPLS-DA score plots and corresponding loading S-Plots obtained from control and (**A**) ALS plasma samples at baseline and (**B**) at follow-up. OPLS-DA loadings S-plots indicate significant lipids species between controls and (**C**) ALS at baseline and (**D**) at follow-up times.

**Figure 2:**
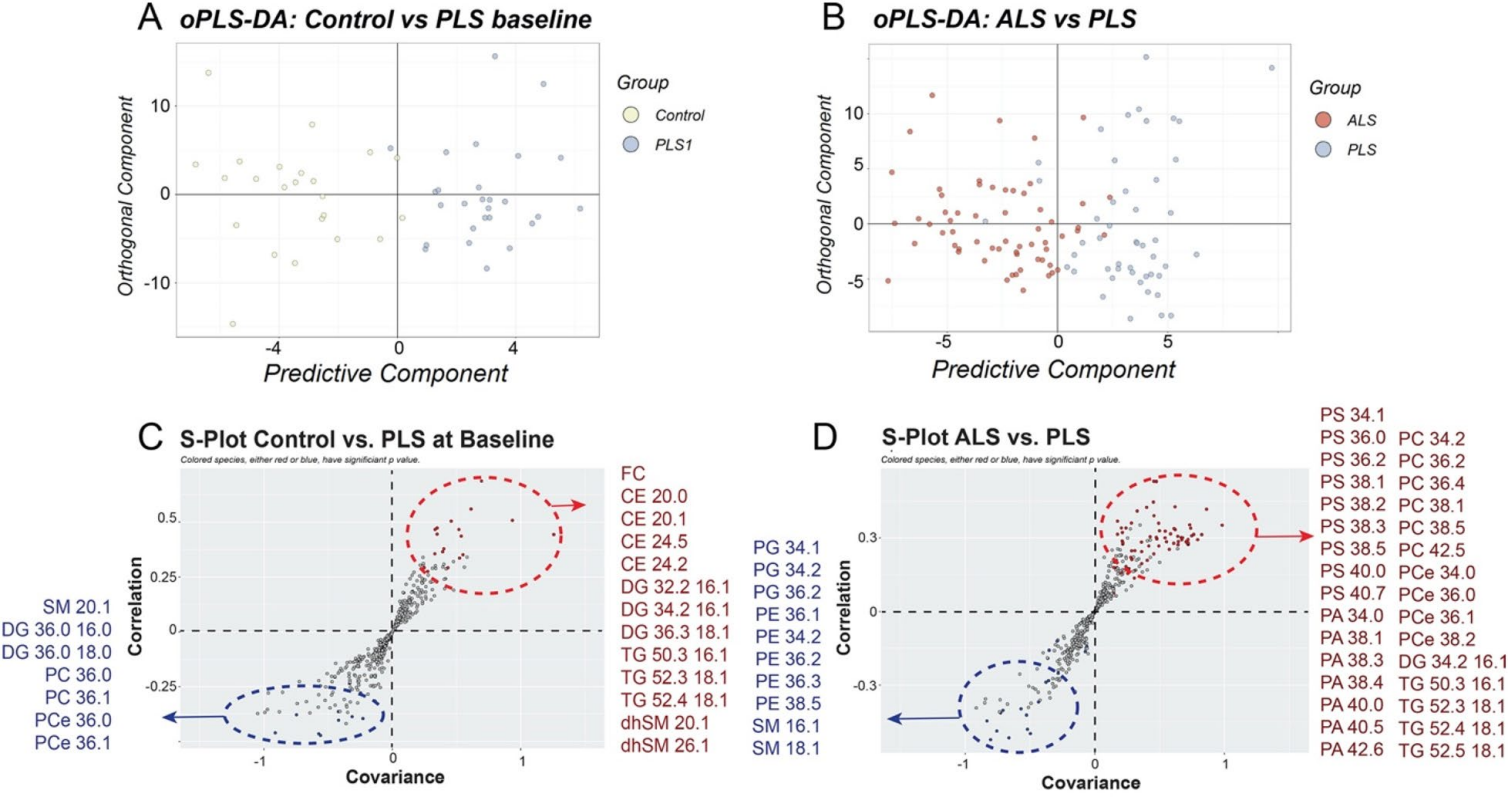
oPLS-DA score plots and corresponding loading S-Plots obtained from (**A**) control and PLS plasma samples at baseline and (**B**) pooled ALS and PLS plasma samples. OPLS-DA loadings S-plots indicate significant lipids species between (**C**) controls and PLS at baseline and (**D**) between all ALS and PLS plasma samples.

To achieve maximum separation between groups, the concentrations of each lipid species was centered, and unit-variance scaled to be analyzed using orthogonal partial least squares-discriminant analysis (oPLS-DA) (Fig. 1A,B). To reject hypothesis that good performances resulted by chance, or overfitting, p values were calculated from the method. For ALS comparisons, all cumulative R^2^Y (explained variation) values were substantially reliable range (Ctrl vs. ALS baseline: 0.712; Ctrl vs. ALS follow up: 0.801) and Q^2^Y (predictive variation) values were all positive^17^. The corresponding scatter plot (S-plots) where covariance (x-axis) and correlation (y-axis) between scores and variables are plotted indicated which lipid species contributed most to the discrimination between groups (Fig. 1C,D). These criteria were also satisfied on comparison between controls and PLS at baseline (R^2^Y: 0.822) (Fig. 2A), although our model diagnostics resulted in negative Q^2^Y values when comparing controls with PLS collected 2-years after, indicating that is not valid to completely separate between these groups. Failure of oPLS-DA to build a valid model could be due to high heterogeneity in our limited number of PLS samples. To overcome this limitation and try to discriminate between both disorders, we pooled together baseline and follow-up samples from each disease and performed an oPLS-DA analysis. This approach separated between groups of disorders with acceptable values (R^2^Y: 0.876) (Fig. 2B). The corresponding S-plots showed the lipid species that contributed the most to the discrimination between PLS at baseline and controls (Fig. 1C), and between pooled ALS and PLS serum samples (Fig. 1D).

**Figure 3:**
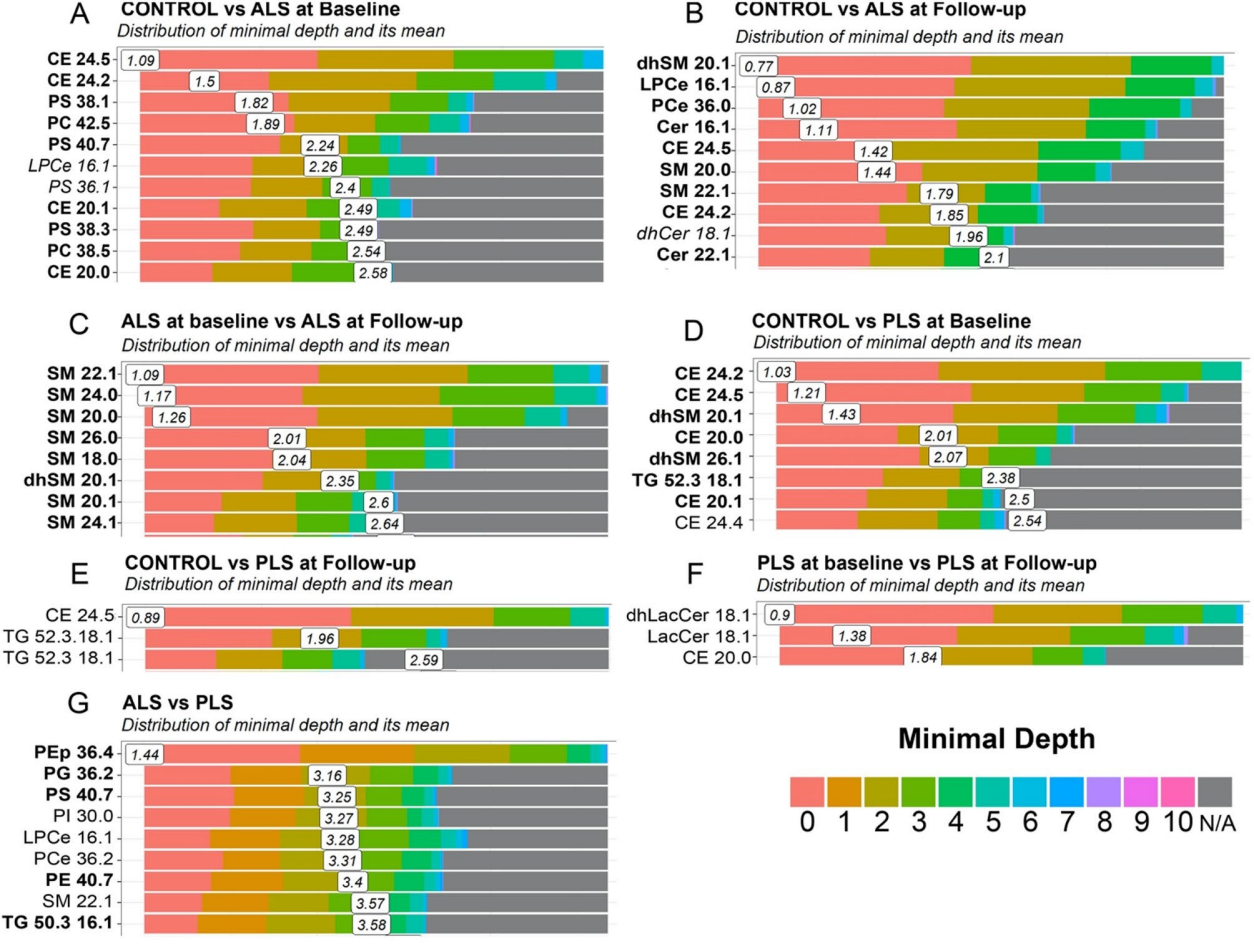
Random Forest plot representing the distribution of minimal depth and its mean for the indicated groups of samples. (**A**) Control versus ALS at baseline, (**B**) Control versus ALS at follow-up, (**C**) ALS at baseline versus ALS at follow-up, (**D**) Control versus PLS at baseline, (**E**) Control versus PLS at follow-up, (**F**) PLS at baseline PLS at follow-up, (**G**) Pooled ALS samples versus pooled PLS samples. Species also identified by oPLS-DA are shown in bold.

We note, however, that although PCA and oPLSDA are the gold standard for binary classification, these discriminant analyses are known to generate models that might overfit the data^18^. On the other hand, machine learning approaches have been shown to be more suited for the analysis of metabolomics data^19^. Thus, as second approach, we used random forest (no. of trees = 5000), a machine learning approach^20^ to select the best performing lipid species per pairwise comparison, based on the lowest mean values for minimum depths in the trees (lower the better) and the frequencies found in trees (higher the better). Minimum depth indicates how early in decision trees a lipid species is involved (Fig. 3). Higher frequencies at lower nodes indicate that some lipid species were effective at classifying the different groups (Supp. Table 2). Specifically, our results indicate alterations in cholesteryl esters CE 24:2 and CE 24:5 are common to both disorders and can discriminate between disease samples and controls (Fig. 3A,B and D,E). Moreover, both ALS and PLS samples present with alterations in sphingolipid species, however the progression of these sphingolipid alterations appears to be more aggressive in ALS plasma samples. Specifically, reductions in various SM species show to significantly contribute most not only when making predictions between ALS and controls samples, but also between ALS plasma collected only 2 years apart (Fig. 3B,C). Similarly, alterations in glycerophospholipid species were only present in ALS cases, some of which (PEp 36:4) were identified as significantly important by RF when discriminating between ALS and PLS samples (Fig. 3G). Finally, changes in TG species containing monounsaturated fatty acids C16:1 and 18:1 (e.g.,TG 50:3/16:1) were only identified in PLS samples as important variables to discriminate between controls and patients (Fig. 3D,E), as well as between both disorders (Fig. 3G).

### Validation and biological interpretations of lipid alterations in ALS and PLS plasma

**Figure 4:**
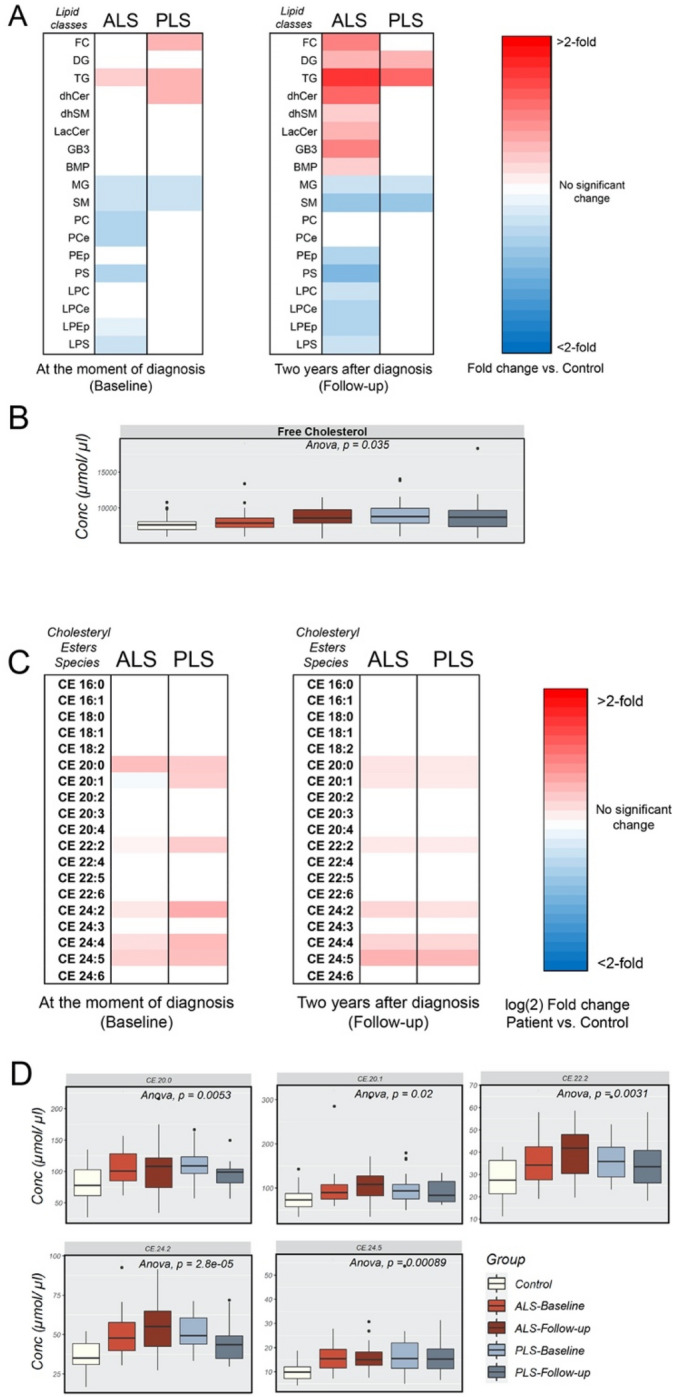
Representation of changes in the main categories of lipids in and cholesteryl esters species in plasma from ALS and PLS patients compared to controls. (**A**) Heat map representation of the most significant fold-changes in the concentration of every class of lipids in plasma from ALS patients compared to controls at the beginning of the study (baseline) and 2 years after (Follow-up). (n = 40 ALS, n = 26 PLS samples and 28 controls analyzed in triplicate. *< 0.05; **< 0.01. T-Test). (**B**) Graph representations of FC concentrations in ALS and PLS plasma. One-way ANOVA. P values are indicated. (C) Heat map representation of the most significant fold-changes in the concentration of cholesteryl ester (CE) species in plasma from ALS and PLS patients compared to controls at the beginning of the study (baseline) and 2 years after (Follow-up) (**D**) Graph representations of the average concentration of CE species in ALS and PLS plasma. One-way ANOVA. P values are indicated.

To validate our results, we first, we compared fold changes in some of the main classes of lipids in plasma from ALS patients at baseline and follow-up times, to those from healthy controls (Fig. 4A and Supp. Fig. 2A). Our data showed that ALS patients present with increases in the concentration of free cholesterol (FC) in plasma 2 years after the beginning of the study compared to controls (Fig. 4B and Supp. Fig. 2A). In the PLS samples, however, elevations in plasma FC showed statistical significance only at baseline (Supp. Fig. 2B). Contrary to unesterified cholesterol, we could not detect any significant changes in the concentration of total cholesteryl esters in neither ALS nor PLS samples (Fig. 4A and Supp. Fig. 2A,B). Nevertheless, we calculated the ratio between CE: total cholesterol, also known as the fractional cholesterol esterification rate (FCE), which is a measure of the rate of cholesterol esterification (Supp. Fig. 2C)^21^. Interestingly, PLS samples showed no difference in FCE rate versus controls over the period of analysis, whereas ALS plasma presented with diminished, although no statistically significant FCE ratios. Given that FCE rates are inversely correlated with the incidence of coronary disease and atheroma formation^21,22^, low FCE ratios in ALS could help explain the incidence of CVD in the disease^23^. This idea is also supported by the significantly higher ratio cholesterol: glycerophospholipids in ALS samples compared to PLS plasma and controls (Supp. Fig. 2D), which has been positively correlated with the risk of atherosclerosis^24^.

While not showing alterations in the total concentration of cholesterol esters (CE), both disorders presented with early increases in particular CE species containing non-essential long and very long fatty acid (VLCFA) acyl chains at baseline (Fig. 4C,D), in agreement with our oPLS-DA results (Figs. 1 and 2). These include CE 24:2 and CE 24:5 species, previously identified by RF as important variables to discriminate between disease and control samples (Fig. 3A,B,D,E and Supp. Table 2). Similar to FC levels, whereas in ALS samples the abnormal levels of these CE species were maintained longitudinally, the increased concentration of some of these CE species tended to stabilize, or even declined over time in PLS plasma (Supp. Fig. 3). Notably, increases in some of CE species containing non-essential VLCFAs could be the result of reduced concentrations of n-3 and n-6 essential fatty acids^25^. In support of this idea, the expression of enzymes involved in the synthesis of non-essential fatty acids was shown to be elevated in ALS and PLS^26^, and increased dietary intake of n-3 fatty acids have been associated with a lower risk of developing ALS^27^.

The levels of glycerolipids were also altered in ALS and PLS plasma (Noted as MG, DG and TG in Fig. 4A and Supp. Fig. 2A,B). For example, the proportion of total monoradylglycerols [1-,2- or 3-acyl-sn-glycerol or monoglycerides (MGs)] was reduced at both baseline and follow-up samples from both ALS and PLS (Supp. Fig. 2A,B), whereas total concentration of diradylglycerols [1,2; 2,3 or 1,3-diacyl-sn-glycerol (DG)] and triradylglycerols [triacyl-sn-glycerols (TGs)] were increased, especially at later stages of both diseases (Fig. 4A and Supp. Fig. 2A,B). Specifically, both ALS and PLS samples showed significant longitudinal decreases in MGs 16:0 and 18:0, although only in PLS were these accompanied by increases in MG species containing oleic (18:1) and linoleic (18:2) acids at baseline (Supp. Fig. 4A,B and E).

**Figure 5:**
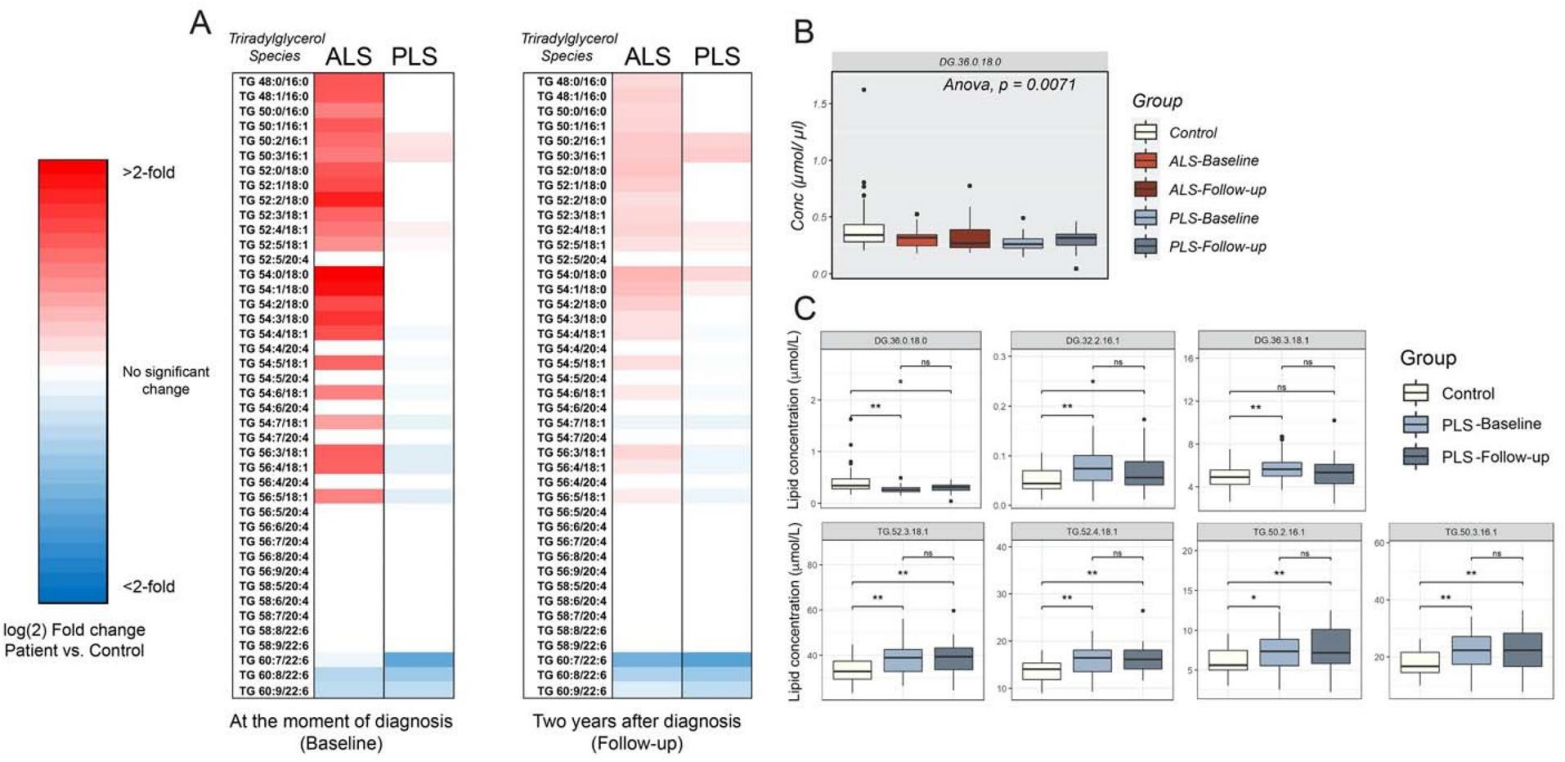
Analysis of TG in plasma from ALS and PLS patients compared to controls (**A**) Heat map representation of the most significant fold-changes in the concentration of Triradylglycerols (TG) species in plasma from ALS and PLS patients compared to controls at the beginning of the study (baseline) and 2 years after (Follow-up) (n = 40 ALS , n = 26 PLS samples and 28 controls analyzed in triplicate. *< 0.05; **< 0.01. T-Test). (**B**) Graph representation of DG species average concentration in ALS and PLS plasma compared to controls. One-way ANOVA. P values are indicated (**C**) Box plot representations of the most significant fold-changes in the concentration of di- and triglyceride species in plasma from PLS patients compared to controls at the beginning of the study (baseline) and 2 years after (Follow-up) (n = 26 PLS samples and 28 controls analyzed in triplicate. *< 0.05; **< 0.01. T-Test).

Both disorders also showed analogous alterations in the concentration of diradylglycerols [1,2; 2,3 or 1,3-diacyl-sn-glycerol (DG)] species in later stages of those pathogenesis (Supp. Fig. 4C,D and F). Specifically, ALS and PLS presented with similar decreases in DG species containing saturated acyl chains (DG 36:0/18:0), although these reductions were only significant in plasma from PLS patients (Fig. 5B). Interestingly, oPLS-DA, but not RF approaches identified DG 36:0/18:0 as an important variable in the characterization of PLS samples (Fig. 2C).

Conversely, the levels of specific DG species containing monounsaturated fatty acids, such as palmitoleic (16:1) and oleic (18:1) acids were significantly increased, especially in PLS plasma (Fig. 5C and Supp. Fig. 4C,D and F). These alterations in the levels of specific DGs were accompanied by increases in TG species esterified with monounsaturated fatty acids, such as TG 50:3/18:1, TG 52:3/18:1, TG 52:4/18:1, TG 54:4/18:1 (Fig. 5A and Supp. Fig. 4F,H), although changes in these specific species were only statistically significant in PLS cases (Fig. 5C and Sup. Fig. 4H).

Although both samples from both disorders showed a significant increase in total TGs, the relative increase in TG species esterified with C16:1 and C18:1 over those containing polyunsaturated fatty acids, was more elevated in PLS when compared to controls and ALS samples (Fig. 5A and Supp. Fig. 4G). This result suggests a potential increase in the de novo TG synthesis and mobilization from adipose tissues. Interestingly, elevations in TGs in ALS have been previously associated with prolonged survival^28^. Therefore, it is possible that the increase in these specific species in PLS, but not in ALS, contributes to the differential aggressiveness of each pathogenesis. In agreement, these TG species were identified by both oPLS-DA and RF approaches when predicting PLS (Figs. 2C and 3D,E,G).

### Alterations in sphingolipids are more dramatic in ALS than in PLS plasma

**Figure 6:**
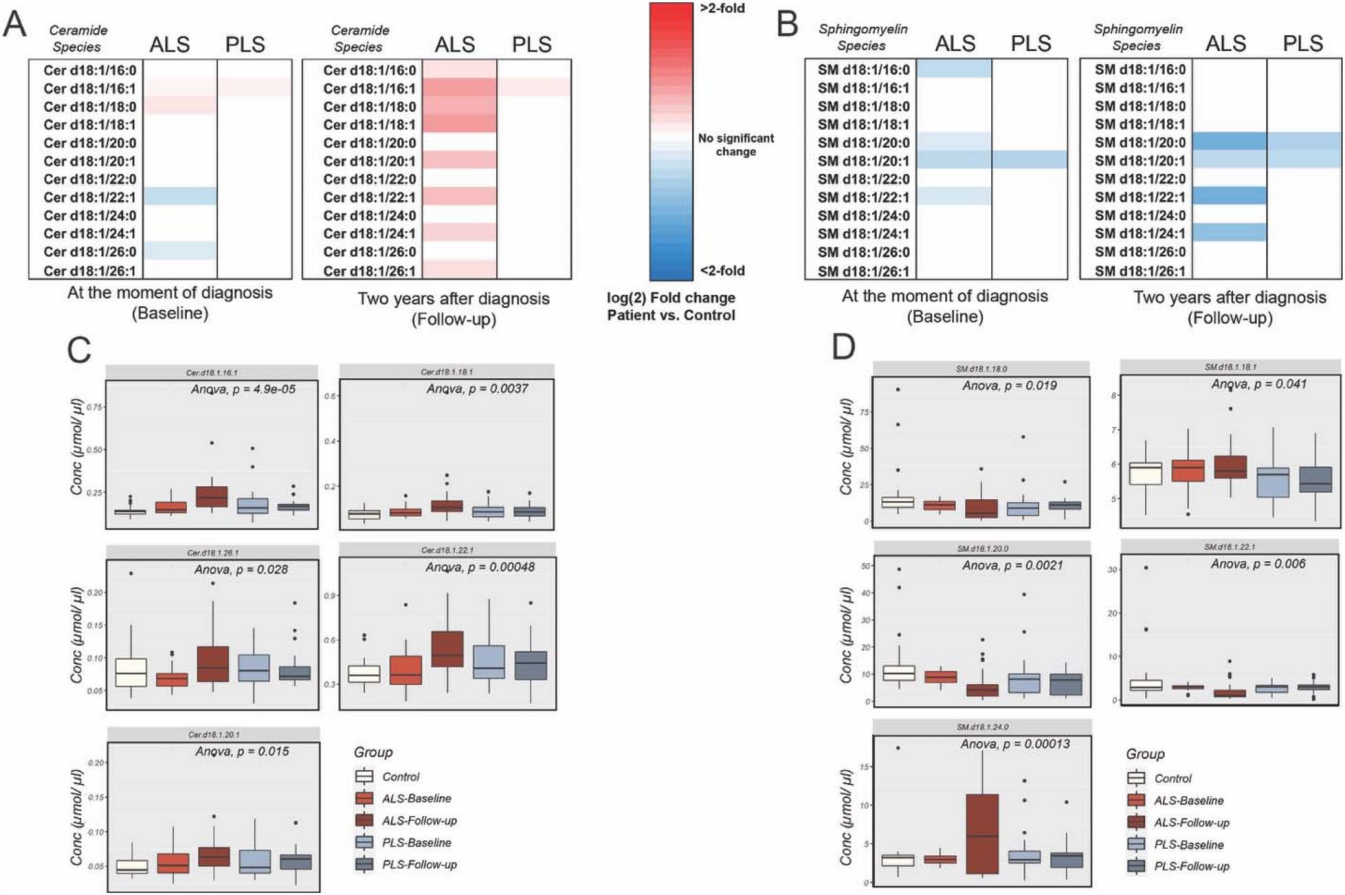
Analysis of ceramide (Cer) and sphingomyelin (SM) changes in plasma from ALS and PLS patients compared to controls (**A**) Heat map representation of the most significant fold-changes in the concentration of (**A**) ceramide and (**B**) sphingomyelin species in plasma from ALS and PLS patients compared to controls at the beginning of the study (baseline) and 2 years after (Follow-up). Graph representations of the average concentration of (**C**) ceramide and (**D**) sphingomyelin species in ALS and PLS plasma. One-way ANOVA. P values are indicated (n = 40 ALS, 26 PLS samples and 28 controls analyzed in triplicate. *< 0.05; **< 0.01).

As for sphingolipid levels, changes in the levels of specific ceramide species (Cer 16:1) were present in both disorders, although the number of species affected and differences in concentration compared to controls were greater in ALS (Fig. 4A and Supp. Fig. 2A). In particular, ALS samples showed longitudinal increases in multiple ceramide species, including those containing very long fatty acids (Cer 22:1 and 26:1) (Fig. 6A,C Supp. Fig. 5A,E), which has been associated to cellular senescence and low cardiovascular fitness^29^.

Our data also showed early and significant reductions in the total content of sphingomyelin (SM) in ALS and PLS compared to controls, with greater reductions at follow-up 2 years later (Fig. 4A and Supp. Fig. 2A,B). In ALS, this initial change in SM was followed by significant increases in other sphingolipids classes at follow-up, such as classes such as lactosylceramides (LacCer), and globosides such as GB3 (Fig. 4A and Supp. Fig. 2A,B).

Among SM species, both ALS and PLS showed early and progressive decreases in the concentration of SM 20:0 and SM 22:1 and 20:1 (Fig. 6B,D and Supp. Fig. 5B and 5D).

Remarkably, these two species were also identified by oPLS-DA and RF as discriminant species between disease and control samples.

Although present in both disorders, these reductions were progressively more severe in ALS compared to PLS (Fig. 6D), and capable of discriminating between ALS samples collected 2 years apart, as previously shown in our RF approach (Fig. 3B). Interestingly, decreases in these species have been previously associated to insulin resistance and lower glucose intolerance^30^, a condition frequently comorbid with motor neuron disorders^31^. In addition to these, ALS plasma samples also presented with progressive increases in SM 24:0 (Fig. 6D, Supp. Fig. 5B,D), which has been shown to positively correlate with increased inflammation^32^.

This progressive higher concentration of several ceramide species in ALS plasma samples suggests that the de novo synthesis of sphingolipids is indeed upregulated in the disease when compared to PLS or control samples, although the contribution of other sphingolipid pathways such as the hydrolysis of SM, glycosphingolipids or gangliosides cannot be ruled out. In fact, this marked alteration in the plasma sphingolipid profile of ALS patients, relative to PLS and controls, is also illustrated in the progressively higher concentration of multiple glycosphingolipids [monohexosylceramides (MhCer) and lactosylceramides (LacCer)], and gangliosides and globosides such as GM3 and GB3 in the plasma of patients with these motor neuron disorder (Supp. Fig. 6). Interestingly, similar sphingolipid signatures have been previously described in the context of pulmonary disorders and lung infections, resulting in altered alveolar surfactant and inflammatory stress^33^.

### Alterations in the levels of glycerophospholipids are only significant in ALS samples

As mentioned above, both ALS and PLS disorders display quite similar longitudinal alterations in the lipidome of plasma samples. Yet, only plasma from ALS patients at both baseline and 2 years after the beginning of the study showed prominent alterations in the levels of major glycerophospholipid classes and species (Fig. 4A and Supp. Fig. 2A), whereas PLS patients did not show any significant alterations in the concentrations of this class of lipids at any point in the analysis.

Analysis of the changes in the main classes of glycerophospholipids in ALS samples showed significant decreases in the total concentrations of 1,2-diacyl-sn-glycero-3-phosphocholine [phosphatidylcholine (PC)], 1-O-alkyl-2-acyl-sn-glycero-3-phosphocholine [phosphatidylcholine ethers (PCe)], 1,2-diacyl-sn-glycero-3-phospho-l-serine [phosphatidylserine (PS)], and 1- or 2-diacyl-sn-glycero-3-phospho-l-serine [lysophosphatidylserine (LPS)], at baseline. Some of these changes progressed into follow-up, when PS, 1-O-alkyl-2-acyl-sn-glycero-3-phospho ethanolamine [phosphatidylethanolamine plasmalogen (PEp)], 1-O-alkyl-sn-glycero-3-phosphocholine [lysophosphatidylcholine ethers (LPCe)], and 1-O-alkyl-sn-glycero-3-phospho ethanolamine [lysophosphatidylethanolamine plasmalogen (LPEp)] were also notably reduced. Interestingly, in follow-up samples, the initially decreased levels of PC and PCe showed significant increases compared to baseline (Fig. 4A and Supp. Fig. 2A).

**Figure 7:**
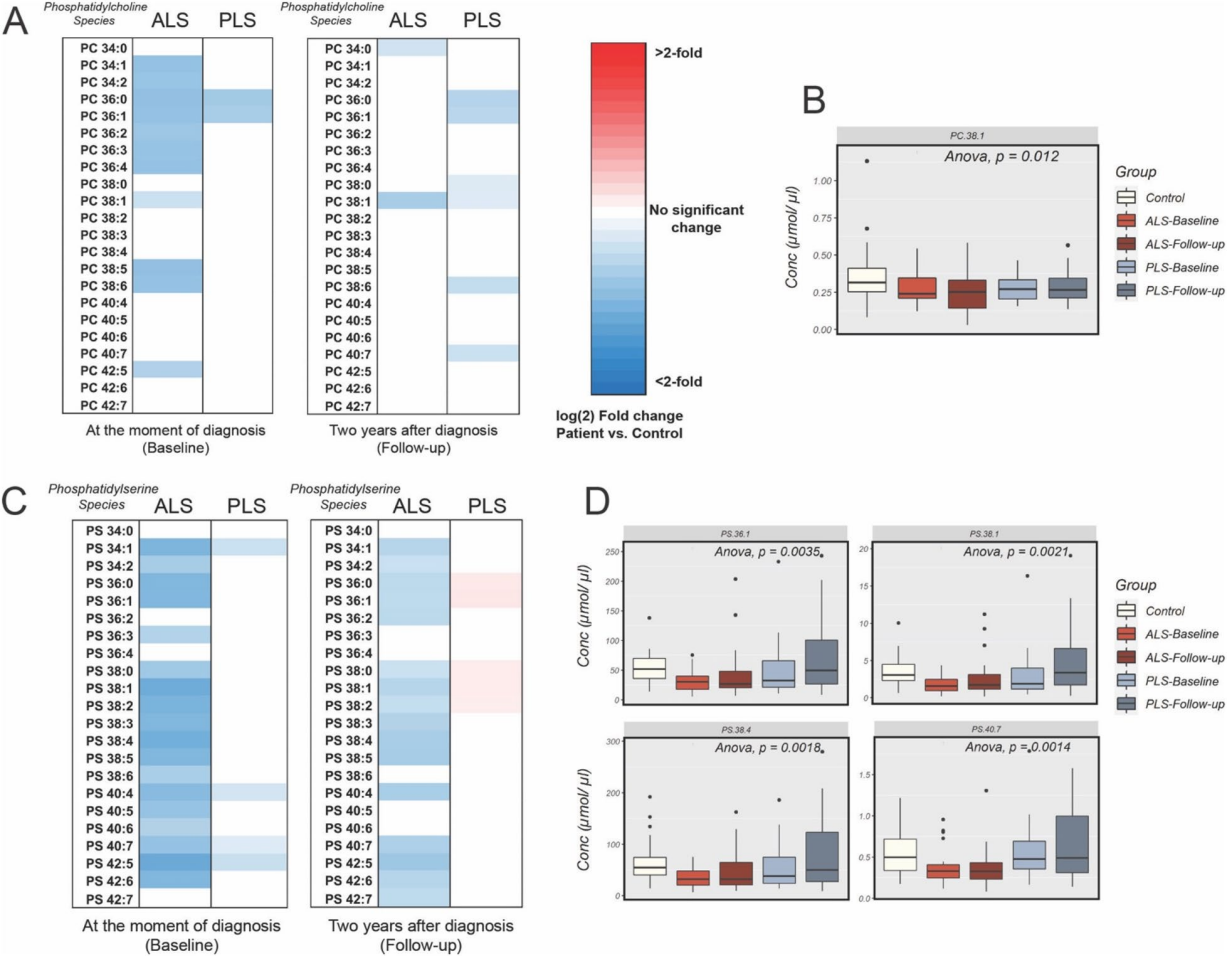
Analysis of PC and PS in plasma from ALS and PLS patients compared to controls (**A**) Heat map representation of the most significant fold-changes in the concentration of glycerophosphatidylcholine (PC) species in plasma from ALS and PLS patients compared to controls at the beginning of the study (baseline) and 1 years after (Follow-up). (**B**) Graph representations of average concentration of specific PC species in ALS and PLS plasma. One-way ANOVA. P values are indicated (**C**) Heat map representation of the most significant fold-changes in the concentration of glycerophosphatidylserine (PS) species in plasma from ALS and PLS patients compared to controls at the beginning of the study (baseline) and 2 years after (Follow-up). (**D**) Graph representations of average concentration of specific PS species in ALS and PLS plasma. One-way ANOVA. P values are indicated (n = 40 ALS, 26 PLS samples and 28 controls analyzed in triplicate. *< 0.05; **< 0.01).

At earlier times in the pathogenesis, ALS samples presented with substantial reductions in several species of PC, PCe, PS, and PEp (Fig. 7 and Supp. Fig. 7). Among all glycerophospholipid classes, PC and PS seem to be particularly and progressively decreased in ALS plasma compared to controls or PLS samples. Specifically, reductions in PS 38:1 and PS 40:7 were identified as significant (Fig. 7 and Supp. Fig. 7) and discriminant between ALS and healthy samples at baseline (Fig. 3A), and PEp 36:4 between ALS and PLS plasma (Fig. 3G and Supp. Fig. 7). While the cause for these specific reductions is unknown, we note that both PEp and PS levels have been shown to be inversely correlated to glucose tolerance and oxidative stress^34^.

Contrary to these glycerophospholipid classes, several species of PE, especially those containing oleic acid and long polyunsaturated acyl chains, were progressively increased in the ALS plasma samples compared to PLS or controls (Supp. Fig. 7E). Remarkably, one of this PE species, PE 40:7 was shown as an important variable when differentiating ALS from PLS samples (Fig. 3G).

Altogether, our data suggest that both disorders result into similar lipid alterations, although these appear to progress faster in ALS that in PLS plasma. For instance, both ALS and PLS plasma present with increases in CE species (e.g., CE 24:5 and CE 24:2) and reductions in specific sphingomyelin species (e.g., SM 20:0) when compared to controls. Yet only ALS samples show a significant progressive reduction in these SM species in the 2 year period of this study compared to PLS. Finally, our data also showed that glycerophospholipid alterations, an in particular in later stages of disease, were essentialy only present in ALS samples, contributing to the discrimination between both disorders.

## Discussion

We have shown here that ALS and PLS samples show similar alterations in plasma lipid profiles that might reflect commonalities between these disorders. In particular, our analysis indicates that changes in neutral lipids are present in both disorders at baseline. For instance, increases in CE with polyunsaturated fatty acids are significantly increased in both diseases. Interestingly, increases in these CE species were also observed in spinal cord tissues of SOD1^G93A^ mice at symptomatic stages^35^.

Moreover, ALS patients show a progressive increase in the concentration of free cholesterol in blood and thus decreases in the CE:FC ratio, an indicator of cholesterol trafficking^36^. Increases in free cholesterol have been previously detected in ALS patients^16,37^. Furthermore, analysis of serum revealed that (25R)26-hydroxycholesterol, the precursor of 3β-hydroxycholest-5-en-26-oic acid, was reduced in ALS patients compared with controls, which indicates altered cholesterol trafficking between glia and neurons and reductions in cholesterol removal^38^. Similar alterations in the concentration of unesterified cholesterol have been observed in animal models where cellular cholesterol uptake is impaired due to mutations in lipoprotein receptors (LDL) or ApoE^39^, suggesting a similar defect in cholesterol internalization in ALS and PLS. Intriguingly, these conclusions could explain previous data that showed that the ε4 allele of ApoE, coding the isoform of the gene with increased capacity of delivering cholesterol intracellularly, presents with a slightly decreased frequency in the ALS population^40^. The conclusions could also explain why the expression of ABCA1 and ABC transporters that promote the efflux of cholesterol have been shown to be highly related to ALS status^41^.

Our data is also in agreement with numerous previous studies showing higher VLDL and LDL/HDL ratios in ALS than in age-matched controls^15,16^. Compared to HDLs, VLDLs and LDLs are enriched in TGs and are poorer in CEs^42^. Thus, our lipidomics data agree with an imbalance of lipoproteins in ALS and PLS patients. Interestingly, the relative increase in TG species esterified with C16 and C18 fatty acids is paralleled by increases in their precursor DG species, which suggests increased de novo TG synthesis and mobilization from adipose tissues. These increases could be the product of the upregulated formation of VLDL particles as a result of increases in fatty acid synthesis^43^. Under these conditions, TG secretion in VLDLs can be saturated and result in hepatic steatosis^43^, a condition frequently found in ALS patients^44^. Furthermore, these TG species have been associated with decreased insulin sensitivity^45^, another condition comorbid of ALS^46^. In support of this idea, several studies have shown increased expression of stearoyl-CoA Delta(9) desaturase (SCD1), one of the enzymes responsible for fatty acid desaturation in the de novo TG synthesis pathway^47^, and of diacylglycerol-O-acyl transferase 2 (DGAT2), one of the two enzymes that catalyze the final reaction in the synthesis of TGs^48^. Moreover, during ALS progression, cells suffer a “metabolic reprogramming” that favors glycolytic metabolism over mitochondrial respiration^49,50^, implying a switch towards the use of fatty acids as carbon sources for ATP production. These metabolic changes, which confer a high risk for cardiovascular disorders, seem to be protective in ALS cases. For instance , hyperlipidemia, diabetes and higher BMI spell out seem to delay ALS onset in human patients^28,51^ and in animal models^52^. Our data shows that, although ALS and PLS patients, show increases in TGs containing C16 and C18, these are only significant and discriminant in PLS plasma (TG 50:3/16:1, TG 52:3/18:1, TG 52:4/18:1). Therefore, it is possible that the increased levels of these specific TGs contribute to the slower progression and lower agressiveness of PLS phenotypes, compared to ALS. .

While many changes were similar between the two diseases, their differential progressions from baseline (at the moment of enrollment in the study) to follow-up (2 years after) indicate that, in PLS, alterations in the levels of specific lipids do not worsen between diagnosis and follow-up, whereas in ALS they do, consistent with the rapid progression of ALS.

For instance, disturbances in the levels of sphingolipids and of the enzymes involved in sphingolipid regulation have been previously described in tissues from ALS mouse models^53,54^ and in cerebrospinal fluid from ALS patients^55^. Interestingly, as in our study, these changes become more significant at more advanced stages of the disease^53^.

Specifically, our data shows significant progressive elevations in short chain ceramides (Cer 16:1, Cer 18:0) and gradual decreases in specific sphingomyelins (SM 20:0 SM 22:1). Interestingly, in contrast to plasma, fibroblasts from ALS patients show increases in the levels of SM^56^. This suggests an impairment in the regulation of SM turnover from cellular membranes, that result in the cytotoxic accumulation of this lipid in ALS cells^56^, and impairments in the regulation of the inflammatory responses^57,58^.

In this study, ALS samples also presented with marked changes in glycerophospholipids that were essentially absent in PLS samples. Interestingly, previous studies have shown an extensive remodeling of glycerophospholipids in ALS, and PCs and PCps in cells from ALS patietns and tissues from SOD1^G93A^ mutant mice at more advanced disease stages, possibly reflecting loss of MNs^59^. In agreement with our data, these studies have shown decreases in PC containg polyunsaturated fatty acids in spinal cord tissues from SOD1 mutant mice^35^. Our data shows that changes in the levels of PEp 36:4 (reduced in ALS) and PE 40:7 (increased in ALS) can discriminate between both disorders.

Interestingly, alterations in the levels of these and other plasmalogen species were identified in fibroblasts from ALS patients as discriminat from controls^60^. These changes were associated to alterations in the composition of mitochondria-associated membranes (MAM) isolated from these cells^60^. MAM is a transient domain in the ER that when formed, recruits multiple enzymes involed in the regulation of lipid metabolism in the cell, such as the synthesis of glycerophosphatidylserine (PS)^61^. Of note, defects in the formation and activation of MAM domains has been observed in cell and animal models of ALS^62,63^. In agreement with this idea, our results show that the levels of PS were progressively reduced in plasma from ALS patients.

Taken together, our data show that changes in the lipid composition of ALS and PLS patients reflect aspects common to both pathologies; however, from a longitudinal perspective, the progression of some of these changes appears to be more exacerbated in ALS than in PLS. However, as mentioned above, given the faster rate of disease progression of ALS compared to PLS and the limited time frame between baseline and follow-up in this study, we cannot exclude the possibility that alterations present only in ALS at follow-up, may as well occur in PLS at a later time points not analyzed here. Under this point of view, our lipidomics data suggest that PLS and ALS are part of a continuum of MN disorders^10^. On the other hand, we can envision an intriguing alternate possibility in which PLS patients actually develop a subtype of ALS but are protected from the aggressive nature of classical ALS by genetic or environmental factors that may buffer (and in some cases stabilize) the aforementioned metabolic alterations, slowing the progression of the disease. Nevertheless, and regardless of whether PLS is a separate entity from ALS or is a condition within the ALS spectrum, our data show that lipidomics analysis can be used to discriminate between ALS and PLS. Our study underscores the use of lipidomics not only as a prognostic indicator to stratify the clinical stages of neurodegenerative disorders, but also as a tool to unveil alterations in specific pathways that could become new targets for future therapeutic trials.

## Materials and methods

### Patients

The patient population studied was derived from a large prospective multicenter study of ALS COSMOS and PLS COSMOS with definite PLS (at least 5 years after symptom onset, providing a well characterized patient population for clinical, neurocognitive, dietary, psychological and environmental features^14,64^. The number of patients with PLS was small (n = 26), 40 patients with ALS were randomly selected within sex and age matched to those of PLS patient population from 355 patients with ALS^9^. For all the participants, blood samples were obtained after overnight fasting. All the patients were longitudinally followed every 3–6 months for 2 years. Plasma was immediately aliquoted and stored at − 80 °C. All biological samples were banked at the Columbia University Environmental Health Science Biorepository.

### Lipidomics

All samples were collected and treated following recently accepted guidelines for the analysis of human blood plasma and/or serum^65^. Lipids were extracted from equal amounts of material (0.2 ml/ sample) by a chloroform–methanol extraction method as described in^66^. Three comprehensive panels, scanning for either positive lipids, negative lipids or neutral lipids (under positive mode), were analyzed for 51 samples. Equal amounts of internal standards with known concentrations were spiked into each extracts (Supp. Table 1). Each standard was later used to calculate the concentrations of corresponding lipid classes by first calculating ratio between measured intensities of a lipid species and that of corresponding internal standard multiplied by the known concentration of the internal standard.

Samples were analyzed using a 6490 Triple Quadrupole LC/MS system (Agilent Technologies, Santa Clara, CA) as described previously^67^. Cholesterol and cholesterol esters were separated with normal-phase HPLC using an Agilent Zorbax Rx-Sil column (inner diameter 2.1 Å–100 mm) under the following conditions: mobile phase A (chloroform:methanol:1 M ammonium hydroxide, 89.9:10:0.1, v/v/v) and mobile phase B (chloroform:methanol:water: ammonium hydroxide, 55:39.9:5:0.1, v/v/v/v); 95% A for 2 min, linear gradient to 30% A over 18 min and held for 3 min, and linear gradient to 95% A over 2 min and held for 6 min.

### Statistical analysis

Intensity signals of each lipid species acquired from the lipidomics analysis were converted to concentration values based on concentration of spiked internal standards of similar ionization efficiencies. The data were first normalized using the NOMIS approach to reduce any systematic variabilities, such as batch effects, as described by Sysi-aho et al.^68^ and implemented in “metabolomics” R package. Then the normalized data were used to draw a PCA graph to confirm the removal of systematic variations as well as to detect any possible outliers that would be subject to biological interpretation. No clustering was observed indicating removal of systematic variabilities by normalization. Samples outside confidence level of 95% were removed from the data set for analysis. The data were used to calculate fold change and *p* value for each comparison made between groups, and these statistics were used to draw heat maps, box plots and volcano plots. “ropls” R package was used for opls-da algorithm^69^. For random forest, “randomForest” and “randomForestExplainer” were used (Paluszynska et al. RandomForestExplainer). Number of trees were set at 5000.

All plasma samples in this study were collected years ago and deposited in the COSMOS repository at Columbia^14^. These samples were provided/gifted by Drs. Mitsumoto and Hupf.

## Supplementary Information

Supplementary Information.
