# Supplementary material for: Lipidomics study of plasma from patients suggest that ALS and PLS are part of a continuum of motor neuron disorders
Source: Sci Rep. 2021 Jun 30;11:13562. doi: 10.1038/s41598-021-92112-3 (PMC8245424; doi:10.1038/s41598-021-92112-3)
Supplement: Supplementary file 1 — Supplementary Information. [file 41598_2021_92112_MOESM1_ESM.docx]

**SUPPLEMENTARY INFORMATION**

**Lipidomics study of plasma from patients suggest that ALS and PLS are part of a continuum of motor neuron disorders**

**Area-Gomez E.^1^*, Larrea D.^1^, Yun T.^1^, Xu Y.^3^, Hupf J.^1,2^, Zandkarimi F.^4^, Chan R.B^3#^., Mitsumoto H.^1,2^***

Departments of ^1^Neurology, ^2^ Institute of Human Nutrition, ^3^Biomarkers Core Laboratory, ^4^Taub Institute for Research on Alzheimer’s Disease and the Aging Brain, ^5^Pathology and Cell Biology, Columbia University Irving Medical Center, New York, NY, USA

**Supplemental Table 1**

| **INTERNAL STANDARD** | **Corresponding Lipid Class** | **Concentration (ug/ul)** |
| --- | --- | --- |
| **IS AcylPG 14:0-28:0** | Acyl PG, NAPE, NAPS | **0.046799614** |
| **IS BMP 28:0** | BMP | **0.015298133** |
| **IS CE C17** | CE | **78.59098931** |
| **IS Cer C17:0** | Cer, dhCer | **0.758320608** |
| **IS Chol d7 b** | Free Cholesterol | **63.78791732** |
| **IS DG 4ME** | diacylglycerols | **0.640874053** |
| **IS dhSM d18:0/12:0** | dihydrosphingomyelins | **2.579623778** |
| **IS DMPC** | AC | **12.34642208** |
| **IS GalCer d18:1/12:0** | MhCer | **1.039897431** |
| **IS LacCer d18:1/12:0** | LacCer | **0.259594347** |
| **IS LPC 13:0** | LPC | **12.34642208** |
| **IS LPE 14:0** | LPE | **0.098349468** |
| **IS LPI 13:0** | LPI | **0.07642123** |
| **IS MG C17** | MG | **0.242952978** |
| **IS PA 28:0** | PA | **0.068072997** |
| **IS PC 28:0** | PC | **12.34642208** |
| **IS PE 25:0** | PE | **8.839285714** |
| **IS PG 12:0/13:0** | PG | **0.446428571** |
| **IS PI 12:0/13:0** | PI | **2.232142857** |
| **IS PS 28:0** | PS | **11.92531331** |
| **IS SM d18:1/12:0** | SM | **13.39285714** |
| **IS Sulf d18:1/12:0** | Sulf | **0.225924621** |
| **IS TG 50:0 d5** | TG | **0.498018035** |

**Supp. Table 1**. Lipid internal standards used in this lipidomics analysis

**Supplemental Figure 1**


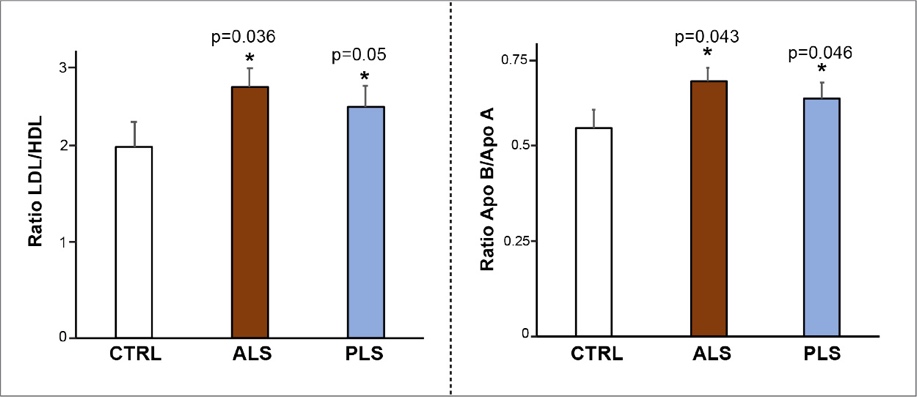


**Supp. Fig. 1.** **Ratios LDH/HDL and ApoB/ ApoA in ALS and PLS serum samples** (Average of n=40 ALS patients; n=28 PLS patients ± S.D. Ancova. p values are indicated on the graph; * p<0.05).

**Supplemental Table 2**


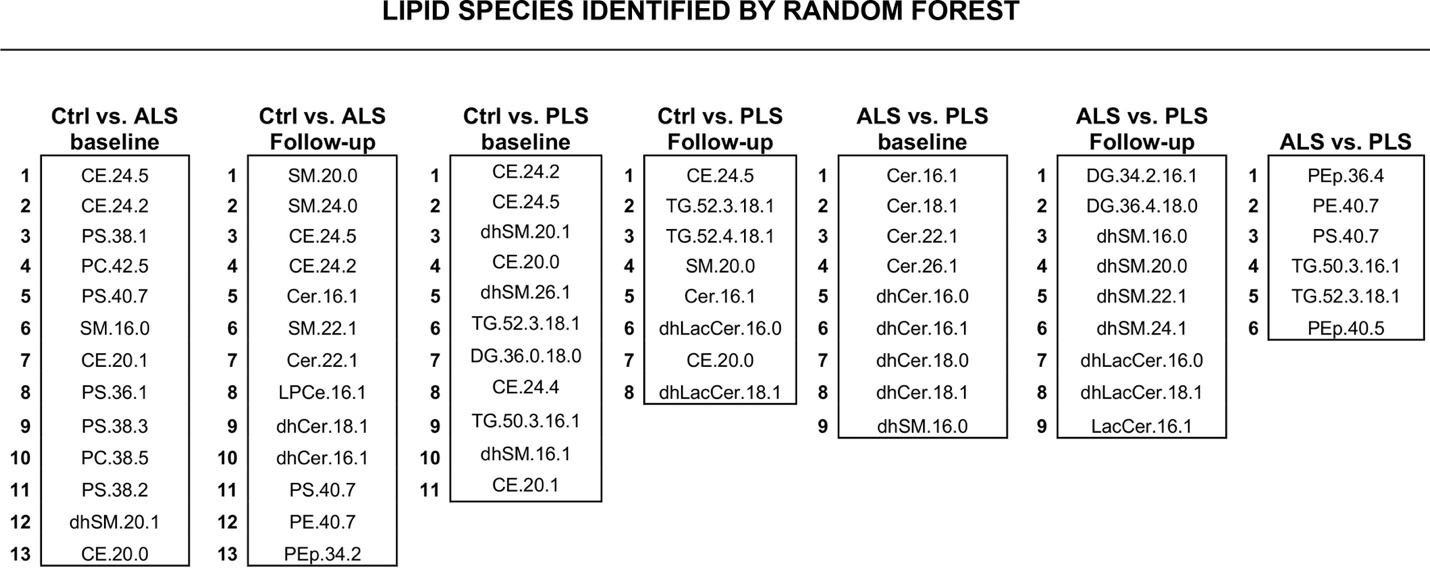


**Supp. Table 1**. Selected species with significant importance scores estimated by random forest classification in the indicated samples.

**Supplemental Figure 2**


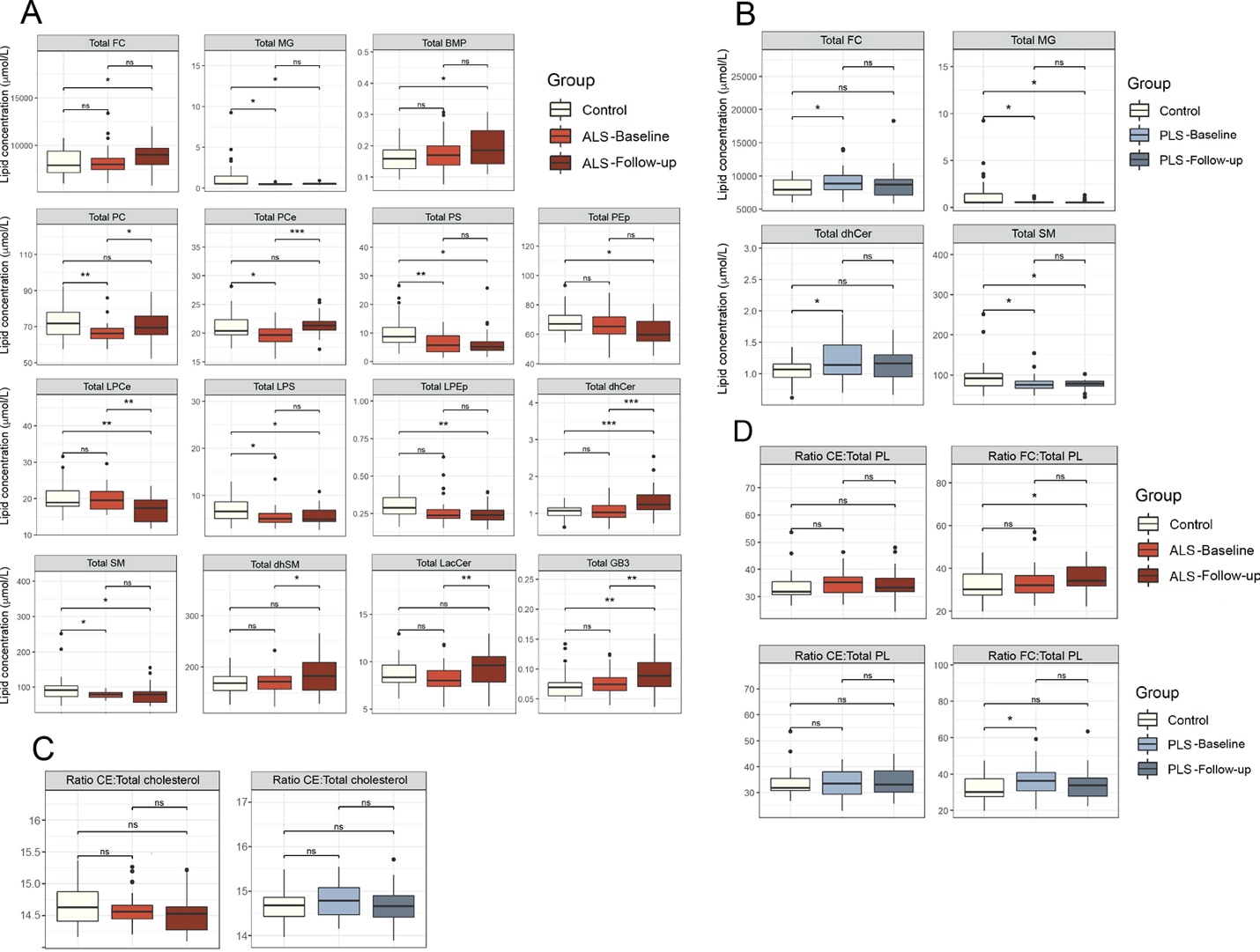


**Supp. Fig. 2. Representation of changes in the main categories of lipids analyzed in plasma from ALS and PLS patients**. Box plot representations of the most significant fold-changes in the concentration of every class of lipids in plasma from (**A**) ALS and (**B**) PLS patients compared to controls at the beginning of the study (baseline) and two years after (Follow-up). (**C**) Ratio cholesteryl ester: Free cholesterol and (D) cholesteryl ester: total glycerophospholipids, and free cholesterol: total glycerophospholipids in ALS or PLS samples. (n= 40 ALS, 26 PLS and 28 control samples analyzed 3 times in triplicate. * <0.05; **<0.01. T-Test).

**Supplemental Figure 3**


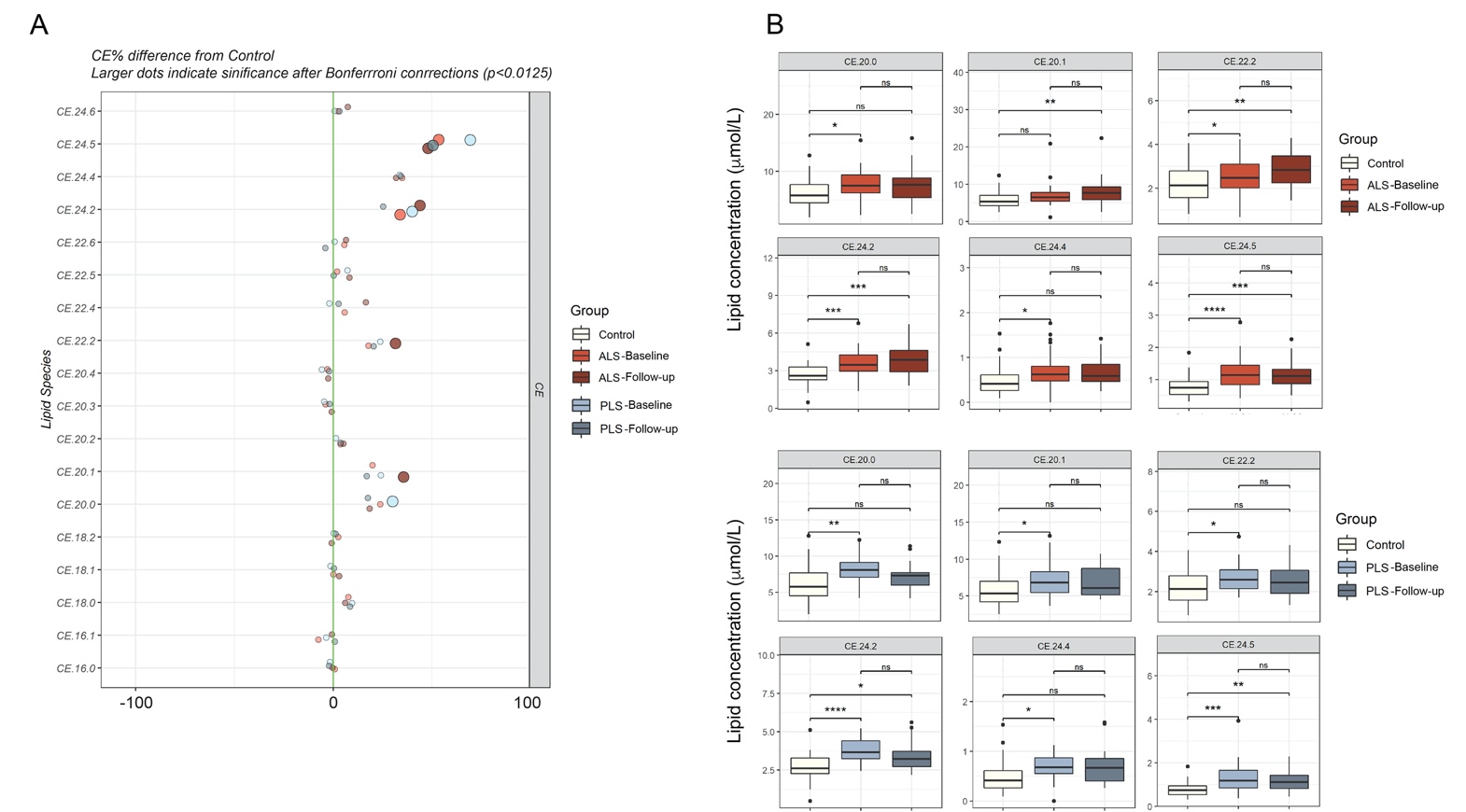


% difference from control levels (green line)

**Supp. Fig. 3. Analysis of cholesteryl esters (CE) in plasma from ALS and PLS patients compared to controls** (**A**) Representation of the percentage difference in the levels of CEs in plasma from ALS and PLS patients compared to controls at the beginning of the study (baseline) and two years after (Follow-up). (**B**) Box plot representations of the most significant fold-changes in the concentration of CEs species in plasma from ALS and PLS patients compared to controls at the beginning of the study (baseline) and two years after (Follow-up) (n= 40 ALS, 26 PLS and 28 control samples analyzed 3 times in triplicate. * <0.05; **<0.01. T-Test).

**Supplemental Figure 4**


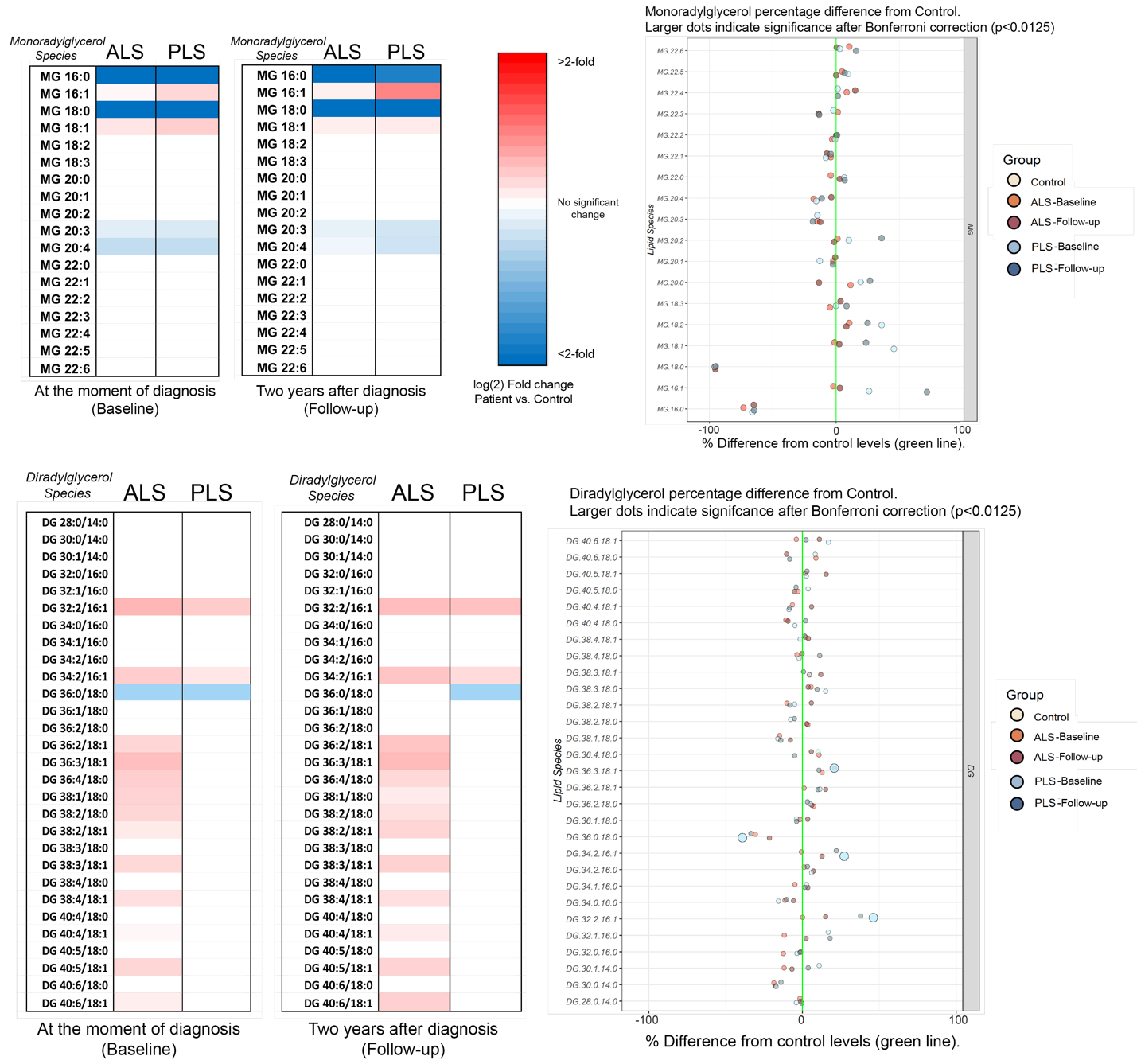


D

C

B

A

**Supplemental Figure 4 continuation**


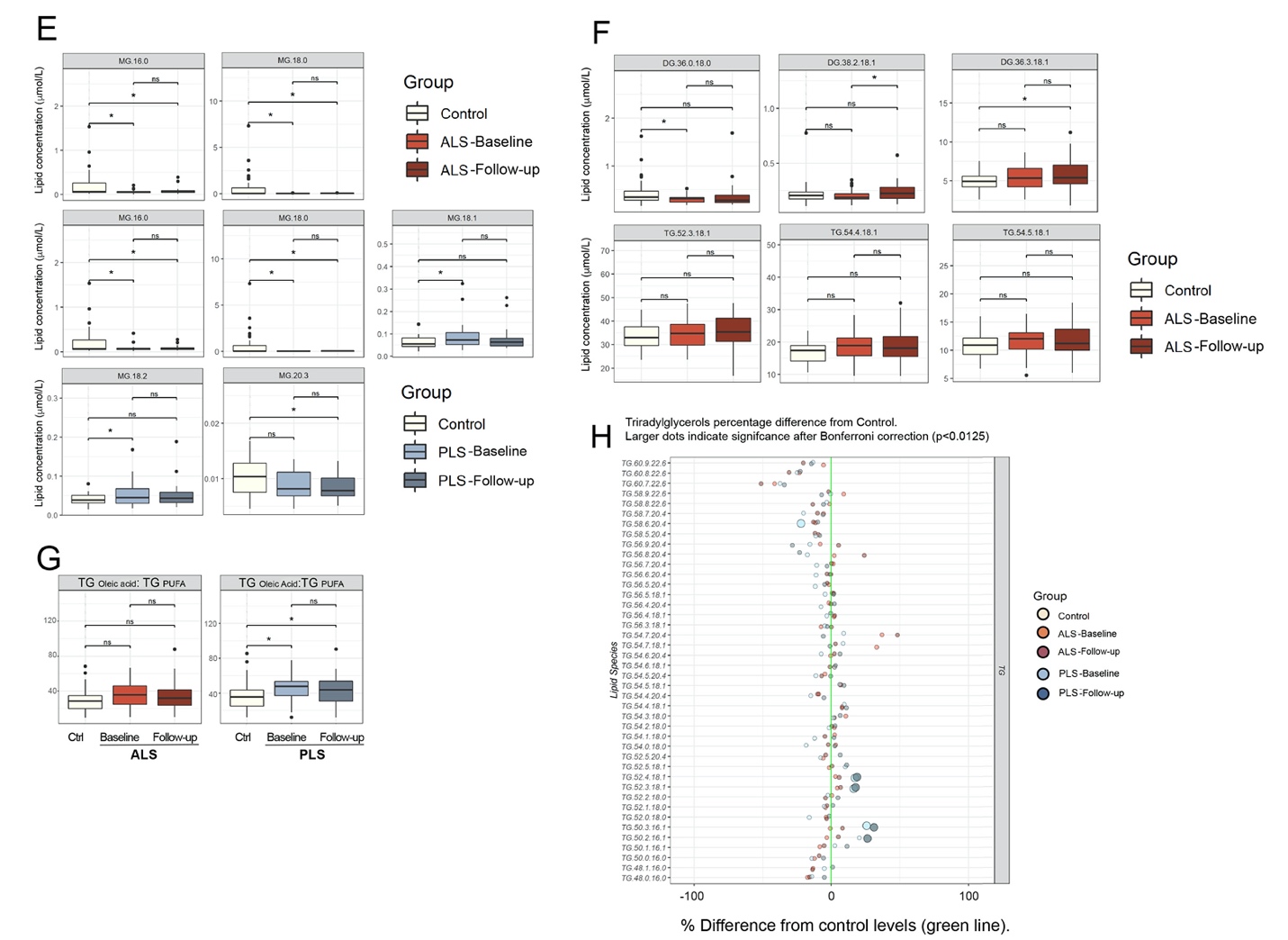


**Supp. Fig. 4. Analysis of mono-, di- and triglycerides in serum from ALS and PLS patients compared to controls** (**A**) Heat map representation of the most significant fold-changes in the concentration of monoradylglycerol (MG) species in plasma from ALS and PLS patients compared to controls at the beginning of the study (baseline) and two years after (Follow-up). (**B**) Representation of the percentage difference in the levels of MGs. (**C**) Heat map representation of the most significant fold-changes in the concentration of diradylglycerol (DG) species in plasma from ALS and PLS patients compared to controls at the beginning of the study (baseline) and two years after (Follow-up). (**D**) Representation of the percentage difference in the levels of DGs. (**E**) Box plot representations of the most significant fold-changes in the concentration of MGs, and (**F**) di- and triglyceride species in plasma from ALS and PLS patients compared to controls at the beginning of the study (baseline) and two years after (Follow-up). (**G**) Box plot representation of the ratios of triglycerides containing oleic acid (C18:1) and triglycerides containing polyunsaturated fatty acids (PUFA) (**H**) Representation of the percentage difference in the levels of TGs. (n= 40 ALS, 26 PLS and 28 control samples analyzed 3 times in triplicate. * <0.05; **<0.01. T-Test).

**Supplemental Figure 5**


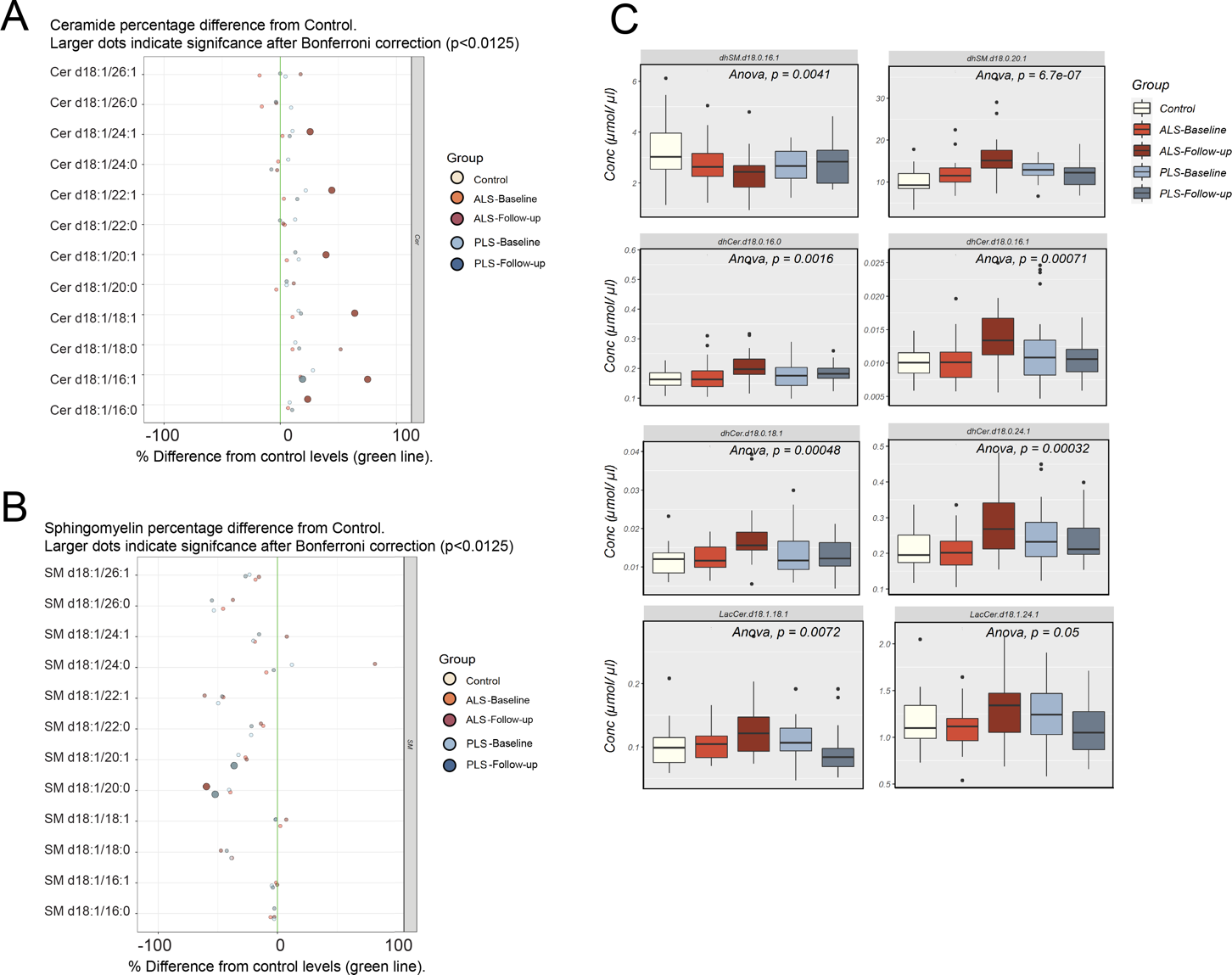


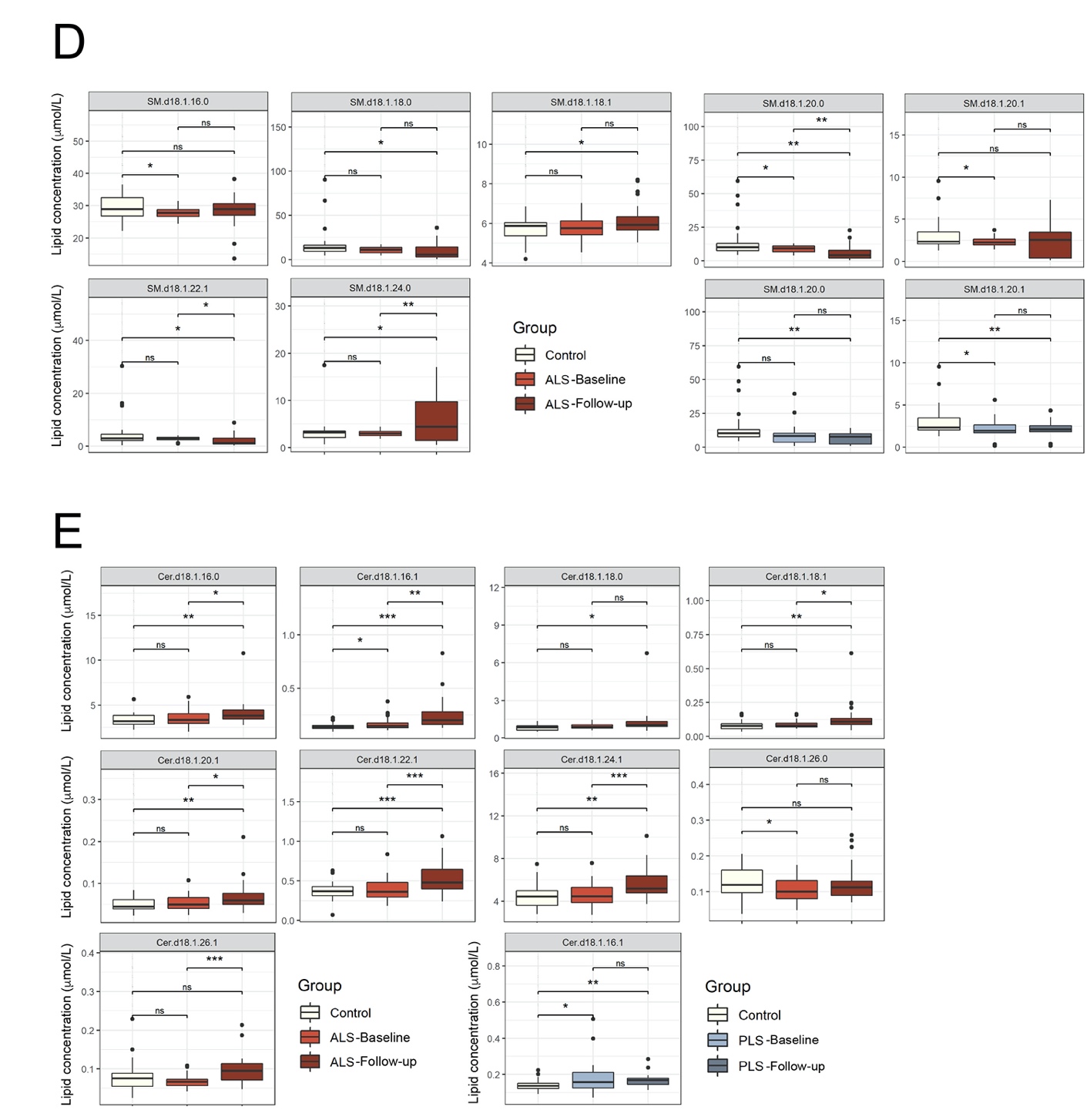
**Supplemental Figure 5 continuation**

**Supp. Fig. 5. Representation of changes in sphingolipids in plasma from ALS and PLS patients**. (**A)** Representation of the percentage difference in the levels of ceramides and **(B**) sphingomyelin species in plasma from ALS and PLS patients compared to controls at the beginning of the study (baseline) and two years after (Follow-up). (n= 40 ALS ALS, 26 PLS and 28 control samples analyzed 3 times in triplicate. * <0.05; **<0.01. T-Test) .

**(C**) Graph representations of the average concentration of specific sphingolipid species in ALS and PLS plasma. One-way ANOVA. P values are indicated (**D**) Box plot representations of the most significant fold-changes in the concentration of sphingomyelin and (**E**) ceramide species in plasma from ALS and PLS patients compared to controls at the beginning of the study (baseline) and two years after (Follow-up). (n= 40 ALS ALS, 26 PLS and 28 control samples analyzed 3 times in triplicate. * <0.05; **<0.01. T-Test)


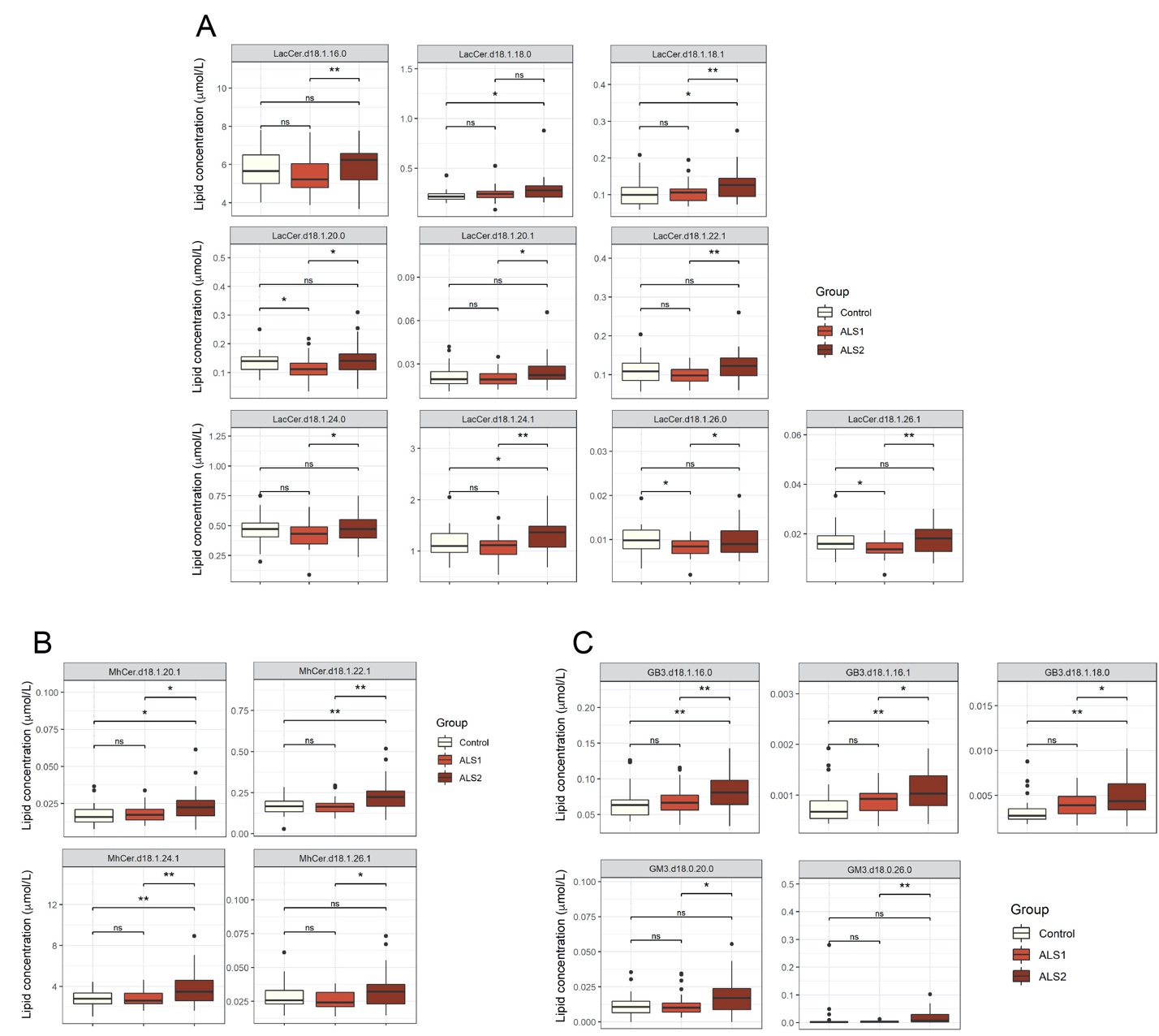
**Supplemental Figure 6**

**Supp. Fig. 6. Representation of changes in sphingolipids in plasma from ALS and PLS patients**. (**A, B, C**) Box plot representations of the most significant fold-changes in the concentration of (**A**) lactosylceramide and (**B**) monohexosylceramide species and (**C**) gangliosides (GB3 and GM3) in plasma from ALS and PLS patients compared to controls at the beginning of the study (baseline) and two years after (Follow-up). (n= 40 ALS and 28 control samples analyzed 3 times in triplicate. * <0.05; **<0.01. T-Test)

**Supplemental Figure 7**


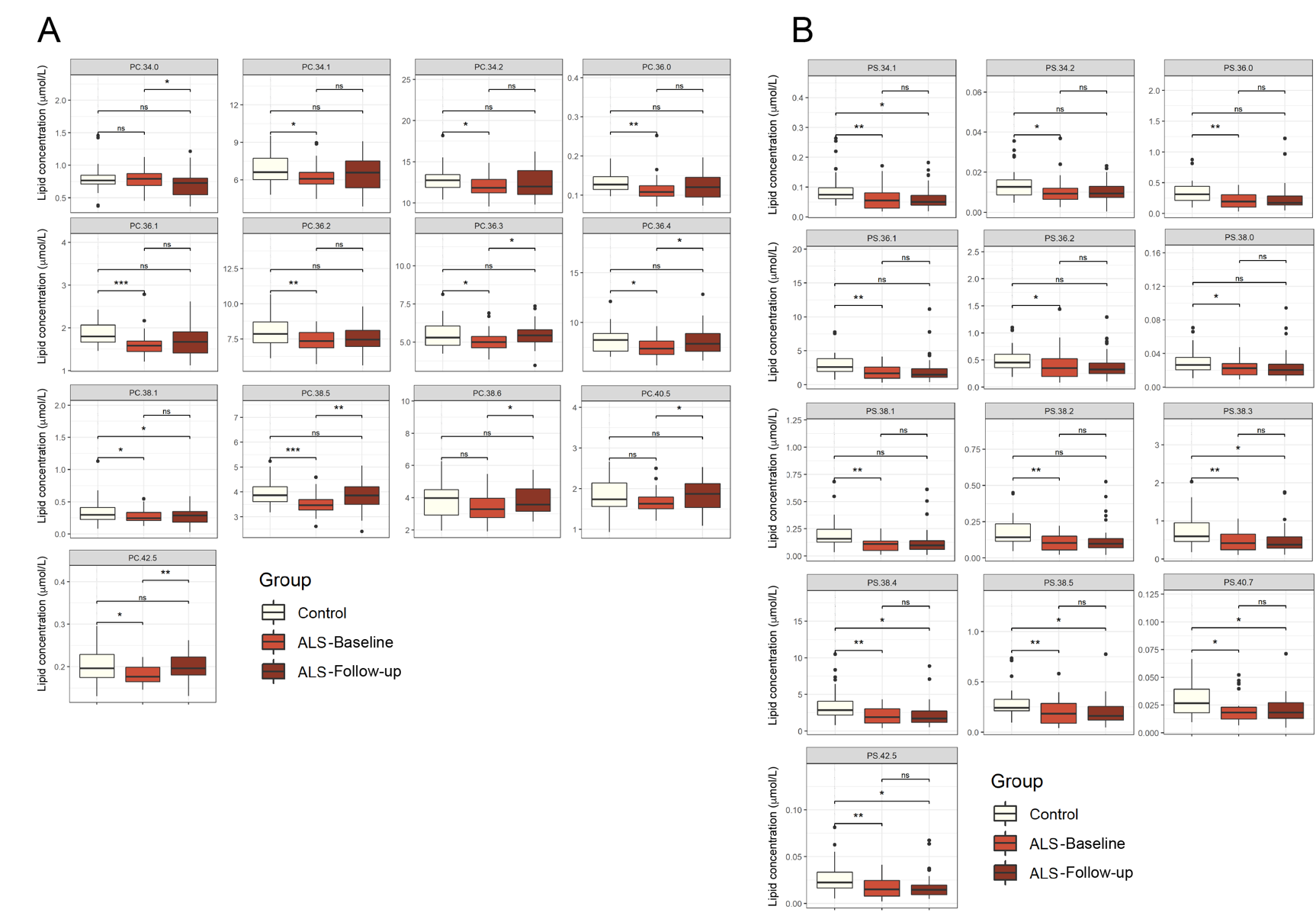


**Supplemental Figure 7. continuation**


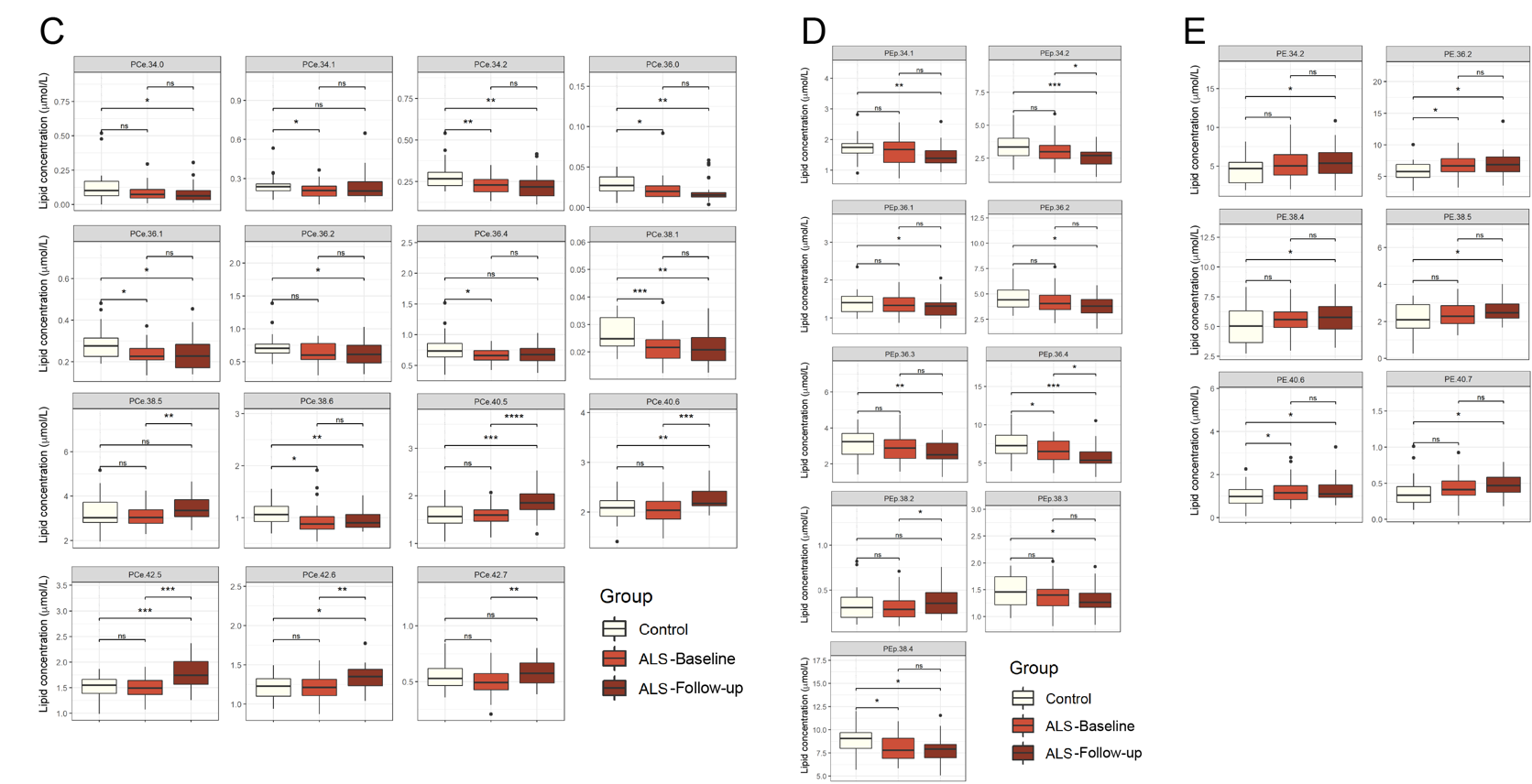


**Supp. Fig. 7. Representation of changes in the phospholipids analyzed in plasma from ALS and PLS patients**. Box plot representations of the most significant fold-changes in the concentration of (**A**) glycerophosphatidylcholine (**B**) glycerophosphatidylserine (**C**) glycerophosphatidylcholine ether (**D**) glycerophosphatidylethanolamine plasmalogen and (**E**) glycerophosphatidylethanolamine species in plasma from ALS patients compared to controls at the beginning of the study (baseline) and two years after (Follow-up). (n= 40 ALS and 28 control samples analyzed 3 times in triplicate. * <0.05; **<0.01. T-Test)
